# Predicting malaria vector distribution under climate change scenarios in China: Challenges for malaria elimination

**DOI:** 10.1038/srep20604

**Published:** 2016-02-12

**Authors:** Zhoupeng Ren, Duoquan Wang, Aimin Ma, Jimee Hwang, Adam Bennett, Hugh J. W. Sturrock, Junfu Fan, Wenjie Zhang, Dian Yang, Xinyu Feng, Zhigui Xia, Xiao-Nong Zhou, Jinfeng Wang

**Affiliations:** 1 State Key Laboratory of Resources and Environmental Information System, Institute of Geographic Science and Natural Resource Research, Chinese Academy of Sciences, Beijing 100101, China; 2 University of Chinese Academy of Sciences, Beijing 100049, China; 3 Key Laboratory of Surveillance and Early Warning on Infectious Disease, Chinese Center for Disease Control and Prevention, Beijing 102206, China; 4 National Institute of Parasitic Diseases, Chinese Center for Disease Control and Prevention, Shanghai, China; 5 World Health Organization Collaborating Centre for Tropical Diseases, Shanghai, China; 6 National Center for International Research on Tropical Diseases, Shanghai, China; 7 College of Geoscience and Surveying Engineering, China University of Mining and Technology, Beijing 100083, China; 8 Global Health Group, University of California, San Francisco, San Francisco, California, United States of America; 9 President’s Malaria Initiative, Malaria Branch, Centers for Disease Control and Prevention, Atlanta, United States of America; 10 School of Civil and Architectural Engineering, Shandong University of Technology, Zibo, China; 11 Jiangsu Center for Collaborative Innovation in Geographical Information Resource Development and Application, Nanjing, 210023, China

## Abstract

Projecting the distribution of malaria vectors under climate change is essential for planning integrated vector control activities for sustaining elimination and preventing reintroduction of malaria. In China, however, little knowledge exists on the possible effects of climate change on malaria vectors. Here we assess the potential impact of climate change on four dominant malaria vectors (*An. dirus, An. minimus, An. lesteri* and *An. sinensis*) using species distribution models for two future decades: the 2030 s and the 2050 s. Simulation-based estimates suggest that the environmentally suitable area (ESA) for *An. dirus* and *An. minimus* would increase by an average of 49% and 16%, respectively, under all three scenarios for the 2030 s, but decrease by 11% and 16%, respectively in the 2050 s. By contrast, an increase of 36% and 11%, respectively, in ESA of *An. lesteri* and *An. sinensis*, was estimated under medium stabilizing (RCP4.5) and very heavy (RCP8.5) emission scenarios. in the 2050 s. In total, we predict a substantial net increase in the population exposed to the four dominant malaria vectors in the decades of the 2030 s and 2050 s, considering land use changes and urbanization simultaneously. Strategies to achieve and sustain malaria elimination in China will need to account for these potential changes in vector distributions and receptivity.

Malaria is a mosquito-borne infectious disease caused by parasites of the genus *Plasmodium*, and is transmitted by female *Anopheles* mosquitoes^1^. Malaria causes a significant burden of disease at the global and regional levels^2^. In the 1940 s, more than 30 million malaria cases were recorded annually in China. After the establishment of the People’s Republic of China in 1949, the Chinese government focused on malaria control, investing significant resources^3^. Following tremendous progress over the past several decades of control, the central government with strong political commitment in 2010 endorsed the National Action Plan for Malaria Elimination (2010–2020), with the goal of national malaria elimination by 2020^4^.

Four dominant malaria vectors occupying distinct ecological niches have been identified in China^3^. *An. sinensis,* an outdoor resting and biting species, is the only known malaria vector in areas above 33 °N latitude and is widely distributed in areas comprised primarily of wet rice cultivation. *An. lesteri*, an indoor resting and biting mosquito and historically the primary vector in areas between 25 °N latitude and 33 °N latitude, is mainly found in low elevation areas and typically breeds in channels, rivulets, sugarcane fields and banana fields. In the areas below 25 °N latitude, *An. minimus* and *An. dirus* are the main vectors. *An. minimus* is indoor resting and biting, while *An. dirus* is usually outdoor resting and biting. The typical breeding sites of *An. minimus* are shaded streams, swamps, pools and seepage in jungle areas. *An. dirus* is often found resting on rock holes, thick grass, bamboo canes and tree roots in the forest.

Scientists have come to a consensus on attributing global warming mainly to atmospheric concentration of man-made greenhouse gases^5^. A number of studies have shown that increases in global temperatures can cause latitudinal and altitudinal shifts in vector distributions, changing the risk of vector borne infectious diseases^6^. Some climate-based models have shown that malaria transmission and burden could expand in temperate zones, while ranges may contract in other regions^7^,^8^. China is expecting a warmer climate in the mid to late 21^st^ century according to future climate predictions^9^. Both predicted annual mean surface air temperature and precipitation are expected to increase, with increases varying between regions. Northern China is expected to be wetter, while southern China is expected to become drier^9^. In addition to mapping the geographical distribution and intensity of malaria transmission^10^,^11^, understanding the distribution of vectors is important for providing strategic and evidence-based information to malaria elimination programs^12^. Furthermore, understanding the possible effects of climate change on the future distribution of dominant malaria vectors will allow programs to adapt vector control strategies including response strategies for imported cases to receptive areas. Here, we assess the impact of various climate change scenarios on the four dominant malaria vectors in China while taking into account mediating factors including land use change and urbanization.

## Results

**Figure 1:**
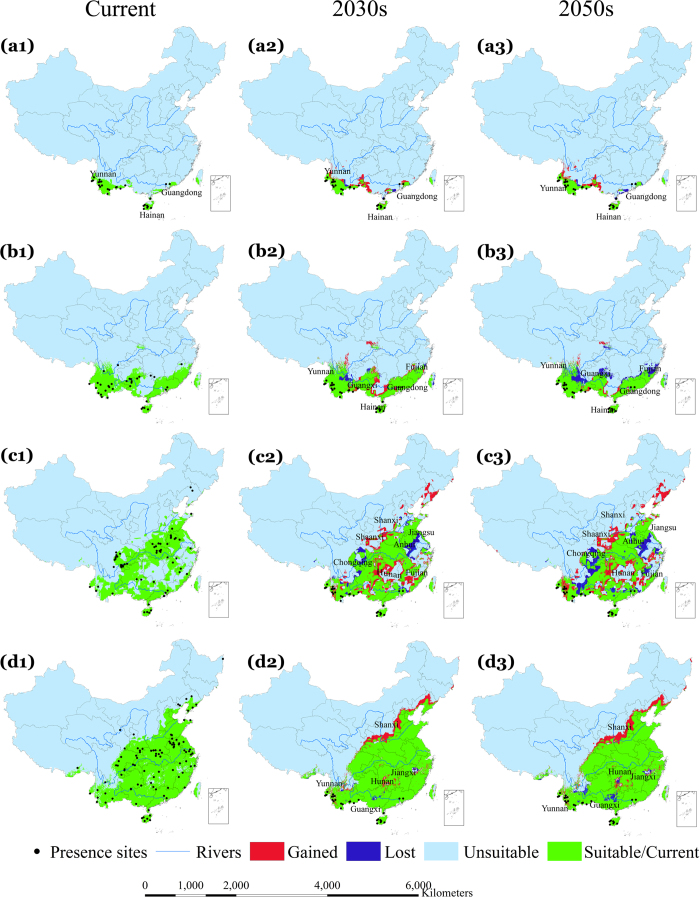
Potential current (suitable and unsuitable) and future (suitable/stable, lost, gained, and unsuitable) environmentally suitable area (ESA) for the four dominant vectors under RCP2.6, the lowest greenhouse gas emission scenario. Row a1–a3 represent *An. dirus*, b1–b3 *An. minimus*, c1–c3 *An. lesteri*, and d1–d3 *An. sinensis*. Future predictions are based on an ensemble of predictions from three general circulation models (BCC-CSM1-1, CCCma_CanESM2 and CSIRO-Mk3.6.0). The second and third columns indicate the 2030 s and 2050 s, respectively. The black dots indicate occurrence localities of the respective malaria vectors. Green shaded areas show stable suitable areas, blue shows lost ESA and red shows gained ESA. All the lost and gained areas were calculated based on the current distribution as the reference. Maps created in ArcGIS 10.2 (Environmental Systems Resource Institute, ArcMap Release 10.2, ESRI, Redlands, California).

**Figure 2:**
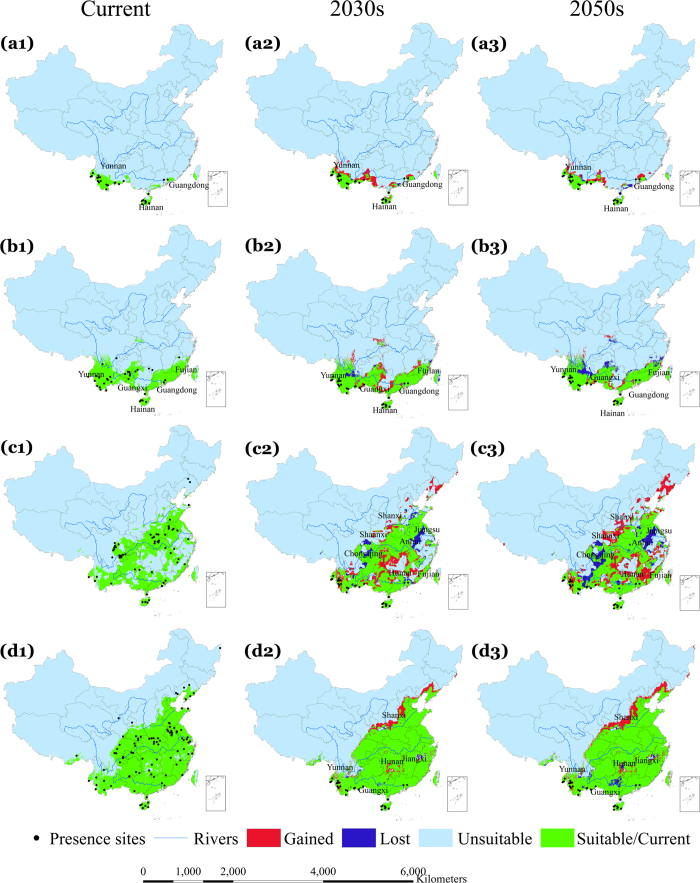
Potential current (suitable and unsuitable) and future (suitable/stable, lost, gained, and unsuitable) environmentally suitable area (ESA) for the four dominant vectors under RCP4.5, the moderate greenhouse gas emission scenario. Row a1–a3 represent *An. dirus*, b1–b3 *An. minimus*, c1–c3 *An. lesteri*, and d1–d3 *An. sinensis*. Future predictions are based on an ensemble of predictions from three general circulation models (BCC-CSM1-1, CCCma_CanESM2 and CSIRO-Mk3.6.0). The second and third columns indicate the 2030 s and 2050 s, respectively. The black dots indicate occurrence localities of the respective malaria vectors. Green shaded areas show stable suitable areas, blue shows lost ESA and red shows gained ESA. All the lost and gained areas were calculated based on the current distribution as the reference. Maps created in ArcGIS 10.2 (Environmental Systems Resource Institute, ArcMap Release 10.2, ESRI, Redlands, California).

**Figure 3:**
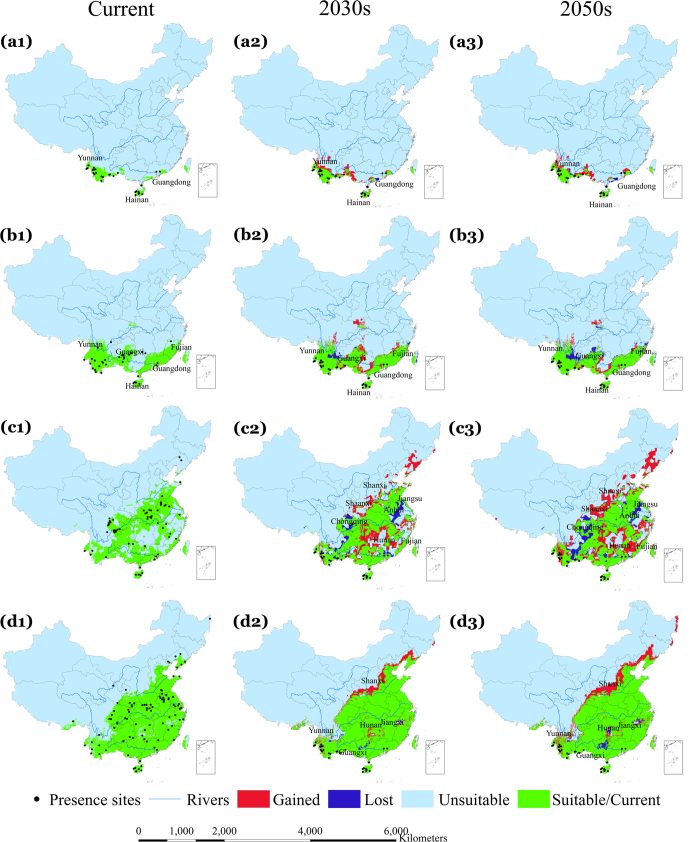
Potential current (suitable and unsuitable) and future (suitable/stable, lost, gained, and unsuitable) environmentally suitable area (ESA) for the four dominant vectors under RCP8.5, the worst greenhouse gas emission scenario. Row a1–a3 represent *An. dirus*, b1–b3 *An. minimus*, c1–c3 *An. lesteri*, and d1–d3 *An. sinensis*. Future predictions are based on an ensemble of predictions from three general circulation models (BCC-CSM1-1, CCCma_CanESM2 and CSIRO-Mk3.6.0). The second and third columns indicate the 2030 s and 2050 s, respectively. The black dots indicate occurrence localities of the respective malaria vectors. Green shaded areas show stable suitable areas, blue shows lost ESA and red shows gained ESA. All the lost and gained areas were calculated based on the current distribution as the reference. Maps created in ArcGIS 10.2 (Environmental Systems Resource Institute, ArcMap Release 10.2, ESRI, Redlands, California).

Figures 1, 2 and 3 indicate the observed presence locations (county centroids) of the four dominant malaria vectors in China and the predicted environmentally suitable area (ESA) for the current, 2030 s, and 2050 s time periods in China under different representative concentration pathways (RCPs, see Methods), RCP2.6 (Fig. 1), RCP4.5 (Fig. 2) and RCP8.5 (Fig. 3). Using external data, the average test area under the curve (AUC) values of our models were 0.977 (*An. dirus*), 0.941 (*An. minimus*), 0.889 (*An. lesteri*) and 0.846 (*An. sinensis*), indicating distinct discriminative ability between suitable and unsuitable areas. The results indicate that the predicted ESA effectively captured the observed malaria vector presence locations according to model-accuracy evaluations (see Methods).

**Figure 4:**
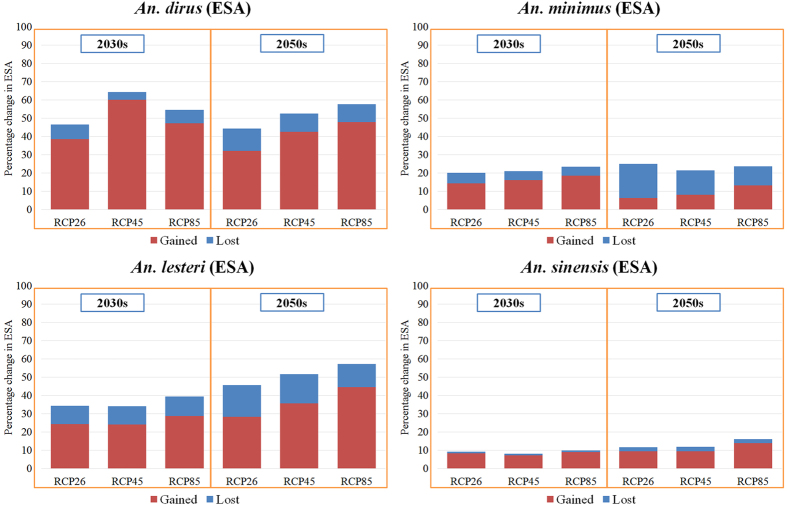
Percent change in estimated gained and lost size of environmentally suitable area (ESA) for four malaria vectors in the 2030 s and 2050 s compared to the present. The projections of changes in ESA were based on an ensemble of simulations from three general circulation models (BCC-CSM1-1, CCCma_CanESM2 and CSIRO-Mk3.6.0) for the 2030 s and 2050 s under three scenarios (RCP2.6, RCP4.5 and RCP8.5).

As depicted in Fig. 4, the effect of future climate scenarios and land use change would increase the potential distribution of the four dominant malaria vectors under all three RCP scenarios in the 2030 s and 2050 s, except for *An. minimus* under RCP2.6 and RCP4.5 (Table 1). Total ESA would increase for the four malaria vector species by larger amounts under the very high emission scenario (RCP8.5) than the medium stabilization scenario (RCP4.5) and the mitigation scenario (RCP2.6) in the 2030 s and 2050 s (Table 1). Further, different general circulation models (GCMs) projected consistent changes in ESA for three species (i.e., *An. dirus, An. minimus,* and *An. sinensis*) in most regions of China (Supplementary Fig. S1), though uncertainty in projections of *An. lesteri* distribution was higher than for other species.

Based on the contribution of related environmental variables to model variation, we have identified important variables (see Environmental variables contributions in Supplementary Notes) for different vectors such as annual temperature range for *An. dirus*, the mean temperature of the coldest quarter for *An. minimus*, and precipitation during the driest quarter and fraction of urban area within grid cell (gurbn) for *An. lesteri* and *An. sinensis*. Since the predicted annual mean surface air temperature and precipitation would increase to the end of this century, with different increase rates among different regions, the projected ESA of the four dominant vectors diverged substantially among different regions over this period. A detailed description of the relationship between vector occurrence and environmental variables can be found in Supplementary Notes.

### Change in ESA of *An. dirus*

Figure 1–a1 indicates that the current predicted ESA for *An. dirus* covers the south-western region of China between 18°–25° N, including Yunnan and Hainan Provinces, as well as a section of coast of Guangdong Province. Predictions using all three GCMs show a consistent increase in ESA for *An. dirus* over the southwest of Guangxi and central parts of Yunnan (Supplementary Fig. S1) Provinces, though large uncertainties in the northwest of Yunnan and northeast of Guangdong Province can be seen from all three GCMs and RCPs. Conversely, a slight decrease in the ESA of *An. dirus* is predicted in Guangdong and Yunnan (Figs 1, 2, 3, a2–3) Provinces. Generally, the ensemble of simulation from three GCMs suggest that the ESA of *An. dirus* will increase in the 2030 s and then decrease in the 2050 s, for all RCPs (Table 1).

### Change in ESA of *An. minimus*

Currently, the ESA for *An. minimus* (Fig. 1–b1) is predicted to cover the south between 18°–33° N including Yunnan, Hainan, Guangxi, Guangdong, and Fujian Provinces. In the 2030 s, the ESA of *An. minimus* is predicted to increase slightly over Guangdong and Guangxi Provinces, and decrease substantially over the same two provinces and Yunnan Province in the 2050 s (under all three RCPs) (Figs 1, 2, 3, b2–3). The models indicate consistent increases in the predicted ESA over the central parts of Guangxi Province and at the border between Guangxi and Guangdong Provinces, while decreases are predicted over the northeastern Yunnan, northern Guangxi, and northeastern Fujian (Figs 1, 2, 3, b1–b3) Provinces, with relatively large uncertainties in other regions including northwestern Hunan Province (Supplementary Fig. S1). With all three RCPs emission scenarios, our model predicted a slight increase in ESA for *An. minimus* in the 2030 s compared to the current distribution, whereas, substantial decreases in ESA are predicted in the 2050 s under RCP2.6 and RCP4.5 (Fig. 4, Table 1). For example, under the RCP2.6 emission scenario, our estimates indicate a predicted increase of 14.3% in the 2030 s, but, decreases of 18.5% in the 2050 s, compared to the current distribution, respectively (Table 1).

### Change in ESA of *An. lesteri*

The current ESA for *An. lesteri* is predicted in the south between 18°–34° N in more than 16 provinces, with a substantially fragmented spatial pattern, especially in the eastern and central parts of China (Fig. 1–c1). The models indicate substantial changes in the ESA of *An. lesteri* by the 2030 s and 2050 s, with considerable variations among GCMs (Figs 1, 2, 3, c1–c3, Supplementary Fig. S1). The northeast and central parts of Hunan, Shaanxi and Shanxi as well as east of Fujian Province are predicted to become environmentally suitable for *An. lesteri* under the three climate projections (Supplementary Fig. S1), while the north of Chongqing, southeast of Anhui, and central Jiangsu Provinces would become unsuitable for *An. lesteri*, with large uncertainties among different GCMs under different RCPs (Supplementary Fig. S1). Specifically, the CCCma_CanESM2 climate model tends to predict a greater increase in ESA than the other two GCMs under the RCP2.6 emission scenario (Supplementary Fig. S4–S6). Furthermore, the models indicate the largest absolute changes in ESA will occur for *An. lesteri* among the four species (Fig. 4). Compared to the current distribution, the relative changes of ESA of *An. lesteri* would increase by 24.5% in the 2030 s and decrease by 28.3% in the 2050 s under the mitigation scenario (RCP2.6). However, for the medium stabilization scenario (RCP4.5) and the very high emission scenario (RCP8.5), the relative changes of ESA of *An. lesteri* would increase gradually from 24.2% and 28.9% in the 2030 s to 35.7% and 44.5% in the 2050 s, respectively (Table 1).

### Change in ESA of *An. sinensis*

The model indicated that the current ESA for *An. sinensis* covers nearly half of China (Figs 1–d1), from the southwest to the northeast of China, coinciding with the most populous regions. All GCM models consistently predicted increasing northern expansion of the ESA for *An. sinensis* (Supplementary Fig. S4–S12), with low uncertainties among different GCM models (Supplementary Fig. S1). Averaged future predictions from the three climatic models for the 2030 s indicated that the ESA of *An. sinensis* could increase in northern and northeastern China. Conversely, a slight decrease in the ESA of *An. sinensis* is predicted in the northeast of Jiangxi and north of Yunnan Province (Figs 1, 2, 3, d2–d3). Model predictions indicate a clear northward shift in the ESA of *An. sinensis* in the 2050 s regardless of emission scenarios (Fig. 4), while central Hunan Province and southern Guangxi Province would be unsuitable (Figs 1, 2, 3, d1–d2). Although the models indicate a consistent slight increase in simulated ESA in the 2050 s compared to the current (Fig. 4, Supplementary Fig. S1) distribution, most of the current ESA are predicted to remain suitable for *An. sinensis* in the 2050 s under the climate change models explored. Under all RCPs explored, our estimates show that the ESA of *An. sinensis* could increase gradually from the 2030 s to 2050 s (Table 1).

### Current and future estimates of the exposed human population

**Figure 5:**
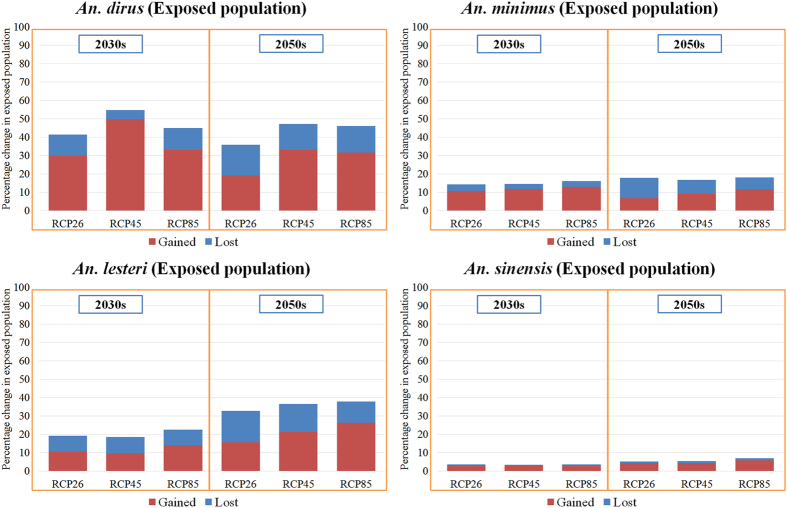
Percent change in estimated gained and lost exposed human population to four malaria vectors in the 2030 s and 2050 s compared to present. The projections of changes in exposed population were based on an ensemble of simulations from three general circulation models (BCC-CSM1-1, CCCma_CanESM2 and CSIRO-Mk3.6.0) for 2030 s and 2050 s under three scenarios (RCP2.6, RCP4.5 and RCP8.5).

The estimates of the percentage change in human population exposed to the dominant malaria vectors under the varying climate scenarios (RCP2.6, RCP4.5 and RCP8.5) derived from an ensemble of simulations from three GCMs (BCC-CSM1-1, CCCma_CanESM2 and CSIRO-Mk3.6.0) are summarized in Fig. 5. Generally, our estimates suggested that the population exposed to lost ESA of all four malaria vectors tends to gradually increase from the 2030 s to the 2050 s. There is a slight decrease in population exposed to the gained ESA of *An. dirus* and *An. minimus* from the 2030 s to the 2050 s. However, our model suggested that population exposed to the gained ESA of *An. lesteri* and *An. sinensis* (Table 1) would increase slightly from the 2030 s to the 2050 s.

Under the RCP2.6 scenario, the human population exposed to *An. dirus* increased on average by 29% in the 2030 s and 19% in the 2050 s, compared with current numbers. Larger increases were observed under the RCP4.5 and RCP8.5 scenarios, both in the 2030 s and 2050 s. Our estimates suggested that the relative increase in exposed population of *An. lesteri* by the 2030 s would be greater under RCP8.5, followed by RCP2.6 and RCP4.5. However, during the 2050 s, the number of people exposed to *An. lesteri* was greatest under RCP8.5, followed by RCP4.5 and RCP2.6, with respect to the current period. The relative change in population exposed was greatest for *An. sinensis* compared with the other three malaria vectors under all three RCPs. In total, under all three RCPs, the population exposed to *An. sinensis* would be much larger than the other three malaria vectors, both in the current and future projections (Supplementary Table S4).

## Discussion

Understanding the current and future distributions of malaria vectors in China is vital for efficient and evidence-based planning for integrated vector control activities to sustain elimination and prevent reintroduction of malaria. Controlling for land use changes and using a range of climate and emission scenarios, together with systematic national entomological surveillance data, this study attempted to predict the current and future distributions of the four dominant malaria vectors. Validation of our models indicated that the predicted ESA accurately captured the current distribution of malaria vector presence. This does not prove a direct causality between environmental variables (climate and land use) and malaria vector distributions, but suggests that climate and land use likely contribute to the overall spatial pattern in China. The agreement between the observed distribution and simulated ESA also suggests our model can be used to project the spatial distribution of malaria vectors in future climate and land use scenarios.

Projections suggest that changes in ESA for the four dominant vectors will occur in future decades, but the size of this change varies according to the climate change scenarios assumed. Compared to the current distribution, results suggest a significant increase in ESA for *An. dirus* in the southwest of Guangxi Province and central parts of Yunnan Province with a slight decrease in Guangdong. For *An. minimus*, the ESA are predicted to increase in the central parts of Guangxi Province and its bordering areas within Guangdong Province, with some decrease in the northern parts of Yunnan, Guangxi and Fujian Provinces. The northeast and central parts of Hunan, Shaanxi and Shanxi Provinces as well as the eastern part of Fujian Province would become suitable for *An. lesteri*, while the north part of Chongqing Province and the southeast part of Anhui Province as well as central Jiangsu Province are predicted to become unsuitable for *An. lesteri.* The ESA of *An. sinensis* are predicted to increase in the north and northeastern regions of China.

The distribution of the predicted ESA for *An. dirus* may be related to the change of potential breeding sites due to deforestation and future land use shifts in China*. An. dirus,* the main malaria vector in parts of Southeast Asia, seems to adapt well to man-made habitats such as orchards and plantations in Myanmar^13^ and Bangladesh^14^. It is possible that the southwest of Guangxi Province and southeast of Yunnan Province could provide suitable habitat in the future. As we incorporated land use in our model, the effect of urbanization on malaria vectors could be captured partly through land use data. Some changes in ESA of *An. lesteri* and *An. sinensis* can be attributed to urbanization (i.e., growth in fraction of urban area within grid cell). This finding was demonstrated by the contribution of the land use variable to niche models of these two species, as well as the relationship between vector occurrence and environmental variables. With urbanization and global climate change, there would be more ESA for *An. lesteri* and *An. sinensis*. However, the impact of urbanization on changes in ESA of *An. dirus* and *An. minimus* would be limited, because bioclimatic variables overwhelmed land use variables for these two species.

Historically, *An. lesteri* was considered a primary vector of malaria in the eastern, central and southern areas of China^15^,^16^. During 1998 to 2001, it was found in 245 counties in 15 provinces, while during 2005 to 2010, it was captured only in 13 counties^17^. In China, *An. lesteri* prefers cool habitats and hibernates through winter in the egg stage in water and moist soil^18^. The projected fragmented spatial pattern in the eastern and central parts of China is consistent with many historical findings that *An. lesteri* was distributed in the foothills of mountain ranges in central parts of China.

Increases in the ESA of *An. sinensis* toward the north and northeastern part of China are likely related to predicted warmer climates in currently colder regions, which would result in more suitable habitats for *An. sinensis* in the future.

The overall predicted increase in ESA for the four dominant malaria vectors represents a potential challenge to China’s ambitious goal of achieving and sustaining malaria elimination. Throughout most of their current geographical distribution, *An. dirus* and *An. minimus* are associated with high malaria prevalence and occurrence of drug resistant *P. falciparum*^19^. The biological specificities of these two vectors, including exophilic behavior, early biting habits and insecticide avoidance, undermine the efficacy of most vector control measures and pose a challenge for achieving and sustaining malaria elimination. Although *An. sinensis* is an inefficient vector, mainly because of its zoophilic habit, it was the primary vector implicated in recent *P. vivax* epidemics in central China due to its high density under suitable conditions^20^. Historically, there have been many local malaria outbreaks caused by imported cases in China including Zhejiang^21^, Hainan^21^, Shandong^22^, and Guangxi^23^ Provinces. Though the distribution of *An. lesteri* has shrunk due to the success of malaria control interventions and improvements in socioeconomic conditions^24^, the ESA for this efficient vector are predicted to increase in the northeast and central parts of Hunan Province with large numbers of imported *P. vivax* cases (7.0% of total country)^25^.

Although climate change is probably a contributing factor to range shifts in ESA for malaria vectors, other factors associated with globalization are also important. Globalization results in explosive growth in the mobility of people and the exchange of goods. With the increasing investment in overseas work and increasing numbers of Chinese persons who are working abroad (e.g., in Africa), imported malaria poses major challenges to malaria elimination in China^26^. Because imported malaria is widely distributed throughout China, the disease could be introduced into malaria-free localities during the transmission season, especially when a large number of cases are clustered in areas in which *Anopheles* species are prevalent. For example, ESA for *An. dirus* are predicted to expand in parts of Guangxi and Yunnan Provinces where imported cases from Africa and South East Asia occur and may be responsible for previous outbreaks^27^. Travel is a potent force in disease spread and emergence with air travel moving human reservoirs or insect vectors great distances in short times.

Like other impact assessments of climate change on species distributions^28^, this study has several limitations. First, our approach did not incorporate the biotic interactions between the four malaria species and other species, such as fish, ditch shrimp^29^ and dragonfly larvae^30^. Predation and competition between species are two common ways biotic interaction and may influence the distribution of mosquitoes^31^. Previous studies indicate that some mosquito species would avoid habitats where competitors are present^32^. Also mosquito species in habitats where predators are present are often absent or in low abundance^33^. Additionally, biotic interactions between other species will be more uncertain due to the spatial distribution of other species that may also change under climate change. Further analysis could combine joint species distribution models and Maxent to estimate the effects of biotic interactions on in the spatial distribution of mosquitoes^29^. Second, we assumed that the association between malaria vector presence and predictor variables based on current or historic data hold true under different climate projection scenarios^28^. This assumption may not hold. The possible evolution of malaria vector characteristics in response to climate change, namely dispersal probability, temperature tolerance (or niche width) and temperature preference (optimal habitat) could also affect the geographical range shifts^34^. Models assumed that the vector species: (1) fully disperse into the projected new ESA; (2) are limited to current ESA; (3) are unable to track the climate change. These assumptions are overly simplistic. One possible solution is to incorporate plausible dispersal scenarios into bioclimatic model projections^35^ to account for the uncertainty of dispersal probability of malaria vectors under climate change. Another issue to consider is that species may evolve to be more adaptable to climate change. Although the impact of climate change on species distributions could be affected by evolutionary changes^36^,^37^, many species evolve slower than climate change^38^ and may not evolve at all. Third, some important variables have not been included in our model due limitations in data availability. For example, changes in farming activities, in particular rampant use of chemical pesticides in rice fields, have created adverse breeding environments, greatly reducing the *An. lesteri* population in some areas^39^. Additionally, estimation of the exposed population to the four malaria vectors in the 2030 s and 2050 s could be biased, as future changes in urban extent were not incorporated. Though there is likely to be some uncertainty in the estimated range shifts of malaria vectors, the performance in the models’ extrapolation capacity according to model-accuracy evaluations (see Model performance in Supplementary Notes) suggest that niche modeling is a reasonable approach^40^ to describe macro-scale patterns of vector distributions.

Although our model predicted that ESA decrease for these four malaria vectors in some geographical regions in 2050 s (Table 1), it is important to consider how the length of the malaria transmission season^41^ may change in the future, as changes in seasonality could increase the number of person-months at risk^1^ in some regions. However, estimating the seasonal patterns of malaria vector persistence using dynamic temperature models^41^ may be better. Future work needs to synthesize dynamic models and species distribution models to provide more detailed information on malaria vector persistence.

Malaria is an extremely climate-sensitive tropical disease, making the assessment of potential change in risk due to projected warming trends one of the most important climate change health questions to address. Our study is the first to assess the potential impact of climate change scenarios on the four dominant malaria vector distributions using three GCMs and maximum entropy species distribution modeling for the 2030 s and 2050 s in China. Given likely limited resources to adequately tackle potential effects of climate change on malaria in the future, this study result will provide the government with the strategic and evidence-based information to adapt and target vector control strategies to achieve and sustain malaria elimination in the future.

## Methods

### Data sources

Vector presence data collected from 62 malaria surveillance sites between 2005 to 2008 were extracted from the national malaria surveillance program database^42^. In addition, a comprehensive and systematic search from CNKI literature system (http://epub.cnki.net/kns/default.htm) of published Chinese language literature was conducted using the following terms malaria vectors, *Anopheles, An. dirus, An. minimus, A. lesteri*, and *An. sinensis* (see Supplementary Methods). The search included mainstream peer-review journals in the fields of parasitology, tropical medicine, biology and entomology. From these searches, 247 articles were identified and the full articles were downloaded. We removed the articles that did not contain information relating to these four malaria vectors occurrence. In order to ensure the quality of malaria vectors presence data, we only kept the administrative unit indicating one or more confirmed occurrence of malaria vectors in a given calendar year. Finally, data from a total of 120 published articles from 2000 to 2010 were compiled (Supplementary Table S1). We recorded the county names and reported Anopheles species. These data were then matched with county level administrative maps in order to assign a location to each presence observation. Based on the National Malaria Surveillance Program, a technical advisory group of 45 experts has been established including malaria epidemiologists, entomologists, and ecologists with more than 5 years of local malaria vector surveillance experiences, 35 from National and Provincial CDC, 10 from University and Institute. All the surveillance results were reviewed by the technical advisory group at the annual program symposium, and all the suspicious samples of Anopheles species (different from recent 2–3 years) were discussed and confirmed using morphological integrated with the molecular methods.

Scenario analysis allows researchers to explore possible future outcomes under climate change and uncertain future interactions between climate and environmental factors like land use^43^. To estimate impacts of plausible future climate conditions (temperature and precipitation) on malaria vectors, we used the newly developed representative concentration pathways (RCPs) under the three general circulation models (GCMs) – BCC-CSM1-1, CCCma_CanESM2 and CSIRO-Mk3.6.0 from Coupled Model Inter-comparison Project 5 (CMIP5). The selected GCMs were chosen because a previous study suggested that these provide the best simulation performance for temperature and precipitation in China^44^. Future climate scenario data were obtained for two time periods (30-y averages): the 2030 s (2020 to 2049) and the 2050 s (2040 to 2069).

The GCMs are mathematical representations of climate system processes including atmosphere, ocean, cryosphere and land surface, and are the primary tools available for simulating the responses of the global climate system to variability in natural and anthropogenic radiative forcing^45^. The RCPs were used as input for general circulation models (GCMs) to simulate future climate trajectories in near and long-term periods. The climate trajectory (i.e., RCP) is used in climate modeling experiments to provide plausible descriptions of how the future climate may evolve with respect to a range of variables, including emissions of greenhouse gases, socio-economic change, land use, and climate change mitigation^43^,^46^,^47^. Three RCPs (RCP2.6, RCP4.5 and RCP8.5) were selected to be representative of three plausible scenarios and included one mitigation scenario (RCP2.6), one medium stabilization scenario (RCP4.5) and one very high emission scenario (RCP8.5)^47^. The RCP2.6 concentration scenario is a representative of mitigation scenarios aiming to limit the increase of global mean temperature to 2°C^48^. To achieve this target, emissions of greenhouse gases would need to decline substantially in order to reach a level of 2.6 W/m^2^ (radiative forcing) by the end of the century. Mitigation strategies including substantial improvement of energy efficiency, replacement of unabated use of fossil fuels by a combination of fossil-fuel use with bioenergy and carbon capture and storage, renewable energy and nuclear power would be used to reduce the cumulative emissions of greenhouse gases by 70% compared to a baseline scenario from 2010 to 2100^48^. The reason we selected different GCMs and RCPs is that prediction results from multiple models and different RCPs could provide uncertainty information for policymakers concerned with impacts and adaptation planning for malaria vector control strategies^49^. Additionally, GCMs can better capture the uncertainty associated with future climate projections and allow for the differences in projections of malaria vector distributions among different GCMs to be assessed^40^.

We used gridded bioclimatic variables (5×5 km), which were based on weather station records obtained from the WorldClim database^50^ to describe the current climate conditions in China. Future bioclimatic variables at the same resolution were obtained from the Climate Change, Agriculture and Food Security climate data portal (http://www.ccafs-climate.org/)^51^. To incorporate the effect of land use change on malaria vectors, a harmonized set of land use scenarios at 0.5°×0.5° resolution (approximately 50 × 50 km) were used to represent current and future (2030 s and 2050 s) land use conditions^52^. Also the impact of urbanization could be captured partly through land use data. The original dataset was resampled to 5×5 km using bilinear interpolation in ArcGIS 10.2 (Environmental Systems Resource Institute, ArcMap Release 10.2, ESRI, Redlands, California) to maintain consistent spatial resolution with the current and future bioclimatic variables. These bioclimatic and land use variables are biologically and statistically plausible for characterizing the four malaria vectors’ ESA (Supplementary Methods).

### Estimation of future population distribution

To estimate current human populations exposed to the dominant vector species, we used a gridded population dataset, available at 1 by 1 km resolution for the year 2010^53^. In order to estimate future population exposed to ESA of malaria vectors, future human population distributions were estimated by multiplying the population in each grid cell by the urban and rural population growth rate. Urban and rural population growth rates were obtained from World Bank population projection dataset from 2010 to 2050^54^. This dataset accounts for predicted future declines in population growth and distinguishes population growth rates in urban and rural areas. Urban extent data were derived from the Defense Meteorological Satellite Program’s Operational Linescan System (DMSP/OLS) nighttime light data, which has been widely used to estimate urban limits^55^,^56^. The DMSP/OLS data we used is a set of composite images in which pixel gives the annual average brightness level in units of 6 bit digital numbers with a spatial resolution of 1 km^57^. We used a threshold of digital numbers >12 to define urban areas based on previous urbanization studies in China^58^,^59^.

### Modelling methods

We used the Maximum entropy (Maxent) species distribution model^60^ with presence-only data and bioclimatic and land use variables (Supplementary Table S2) to predict the current and future potential ESA for the four dominant malaria vector species. Maxent models, using presence only data, have been widely used for modeling species distributions, and have been shown to have excellent predictive performance compared to other structured decision making models, including those using presence-absence data^61^,^62^. Further, Maxent models have been used to project species distributions under future climate change conditions^63^,^64^. The principle of Maxent models is to estimate the probability of species presence by finding the distribution of the maximum entropy (i.e., closest to uniform), with constraints imposed by the observed spatial distributions of the species and environmental data^60^. As only county level (polygon level) vector presence data were available, whereas the explanatory variables were available at 5 × 5 km, we used an approach termed “point sampling” to model the data. This approach has been previously used to generate species distribution predictions at fine resolutions from coarse-scale presence records (e.g., county level presence records)^65^. Point sampling involves assigning the presence data a random location within each county. Explanatory variables are then extracted at that point and assigned to the presence data for modeling with Maxent. To incorporate the uncertainty introduced by the random assignment of presence location within each county, we repeated this process 100 times and calculated the mean prediction value at each pixel. To get more information of assumptions, limitations and evaluation of the point sampling approach, see Supplementary Notes.

We then projected the spatial distribution of each species ESA over two time periods (decades of the 2030 s and the 2050 s) by applying the models to future climate and land use scenario data. We also compared the ESA distribution projected by three different GCMs to investigate variation in predictions under different GCMs (see Supplementary Notes and Supplementary Fig. S12). In order to map the distribution of ESA for dominant vectors, we used a 10th percentile training presence threshold based on the mean value of 100 random point sampling processes to convert continuous presence probability maps into binary ESA maps (suitable and unsuitable)^64^. Current and future exposed populations were estimated by overlaying the binary ESA maps of each species on a gridded population distribution map. All maps were created in ArcGIS 10.2 (Environmental Systems Resource Institute, ArcMap Release 10.2, ESRI, Redlands, California).

### Validation and evaluation of models

To evaluate model performance, we split the data into two parts randomly: training and validation datasets. In this study, 75% of the presence data were randomly selected to act as training data, with the remaining 25% acting as validation data^64^,^66^. In order to take into account uncertainty introduced by training and validation set splits, 30 models for each species were produced by 30 replicate runs of the Maxent model. All the data were used to make the final predictions.

The most often reported measure of Maxent output is the threshold-independent assessment using the area under the curve (AUC) metric of the Receiving Operator Curve (ROC). The ROC was used to investigate the trade-off between sensitivity and specificity over a range of classification thresholds^61^. While the AUC evaluates the ability of models to correctly predict a higher probability of occurrence where species are present than where they are absent^61^. The AUC value has a range between 0 and 1, 0.5 indicates random prediction, and higher values correspond to better models.

### Selection of predictor variables

We selected bioclimatic variables (Supplementary Table S2) that met three criteria^40^: those that (1) are statistically important for fitting the anopheles presence data, (2) are biologically important for anopheles survival, and (3) do not display collinearity with other bioclimatic variables. Variables were compared using univariate models and AUC values. For variables with high predictive accuracy but high collinearity with other variables, we selected the variables that produced the highest AUC when included in a univariate model, excluding the most correlated variables (e.g., mean temperature in the coldest month and mean minimum temperature) (Pearson’s correlation coefficients>0.75). We also inspected the relationship between the probability of anopheles presence and bioclimatic variables in a response curve plot (Supplementary Notes and Supplementary Fig. S2). This process led to a final set of 13 predictor variables (Supplementary Table S2), which were used in the final model. All the land use variables were selected for modeling distributions of all four dominant species, due to their importance for malaria vector distributions and low collinearity with other variables.

## Additional Information

**How to cite this article**: Ren, Z. *et al.* Predicting malaria vector distribution under climate change scenarios in China: Challenges for malaria elimination. *Sci. Rep.* **6**, 20604; doi: 10.1038/srep20604 (2016).

## Supplementary Material

Supplementary Information

## Figures and Tables

**Table 1:** Percentage gained and lost in enviromentally suitable area (ESA) and relevant exposed human population for four malaria vectors in the 2030 s and 2050 s as compared with the current.

Species	RCPs	ESA (thousand square kilometres)				Exposed population (millions)			
		2030 s		2050 s		2030 s		2050 s	
		Gained	Lost	Gained	Lost	Gained	Lost	Gained	Lost
*An. dirus*	RCP2.6	38.5	8.0	32.2	12.1	29.9	11.7	19.3	16.7
	RCP4.5	60.2	4.2	42.7	9.9	50.0	4.9	33.3	14.0
	RCP8.5	47.3	7.2	48.0	9.7	33.3	11.7	31.8	14.4
*An. minimus*	RCP2.6	14.3	5.8	6.5	18.5	10.9	3.4	6.9	11.1
	RCP4.5	16.2	4.8	8.1	13.4	12.0	2.6	9.2	7.7
	RCP8.5	18.6	5.0	13.2	10.5	13.0	3.1	11.8	6.4
*An. lesteri*	RCP2.6	24.5	9.8	28.3	17.4	10.6	8.7	15.8	17.0
	RCP4.5	24.2	9.9	35.7	16.1	9.8	8.9	21.5	15.2
	RCP8.5	28.9	10.6	44.5	12.8	14.2	8.4	26.3	11.7
*An. sinensis*	RCP2.6	8.4	1.0	9.5	2.3	3.3	0.4	4.4	1.0
	RCP4.5	7.4	0.9	9.6	2.4	3.0	0.5	4.4	1.2
	RCP8.5	9.1	0.9	14.0	2.1	3.4	0.4	5.9	1.1

All the estimations were based on an ensemble of simulations from three general circulation models (BCC-CSM1-1, CCCma_CanESM2 and CSIRO-Mk3.6.0) under RCP2.6, RCP4.5 and RCP8.5 climate scenarios.
