# Supplementary material for: Predicting malaria vector distribution under climate change scenarios in China: Challenges for malaria elimination
Source: Sci Rep. 2016 Feb 12;6:20604. doi: 10.1038/srep20604 (PMC4751525; doi:10.1038/srep20604)
Supplement: Supplementary Information [file srep20604-s1.pdf]

Supplementary information for

Predicting malaria vector distribution under climate change scenarios in China: Challenges for malaria elimination

Zhoupeng Ren, Duoquan Wang, Aimin Ma, Jimee Hwang, Adam Bennett, Hugh J. W. Sturrock, Junfu Fan, Wenjie Zhang, Dian Yang, Xinyu Feng, Zhigui Xia, Xiao-Nong Zhou & Jinfeng Wang

This document includes:

-Detailed Information of Datasets
  Mosquito presence data
-Supplementary Notes
  Environmental variables contributions
  Relationship between vector occurrence and environmental variables
  Consistence among different GCM models
  Assumptions, limitations, and evaluation of point sampling approach
-Supplementary References
-Supplementary Tables S1-S4
-Supplementary Figures S1-S13

Detailed Information of Datasets

Mosquito presence data

The presence records for malaria vectors in this study came from an exhaustive and systematic search of formal as well as informal publications. Records were available from six years (2005-2010) of longitudinal surveillance data in the national malaria surveillance program in China. Based on results from the national malaria surveillance program during 2005-2010, formal and/or informal publications related to the occurrence of dominant vectors including *An. dirus* [1-5], *An. minimus* [2,3,5-33], *An. sinensis* [34-88], *An. lesteri* [20,24,89-120], were searched for and confirmed by a technical advisory group of *Anopheles* experts including malaria epidemiologists, entomologists and ecologists. We also searched formally published literatures from 2000 to 2010. From these searches, 247 articles were identified and the full articles were downloaded. We removed the articles that did not contain information relating to these four malaria vectors occurrence. Records of the presence of the dominant malaria vectors at a particular site and survey date were entered into the database so that information collected at different times from a locality was documented. In order to ensure the quality of malaria vectors presence data, we only kept the administrative unit indicating confirmed more than three times occurrences of malaria vectors in a given searching period. Finally, data from a total of 120 published articles from 2000 to 2010 were compiled (Supplementary Table S1). We recorded the county names and reported Anopheles species. These data were then matched with county level administrative maps in order to assign a location to each presence observation.

A total of 27 (*An. dirus*), 33 (*An. minimus*), 59 (*An. lesteri*) and 95 (*An. sinensis*) county level presence records from 2000 to 2010 were identified. For visualization purposes, the centroid of each county was used to map current presence data (Supplementary Fig. S2).

Supplementary Notes

Environmental variables contributions

The relative contributions of the environmental variables are shown in Supplementary Fig. S2. Based on the Maxent model's internal jacknife test of variables importance, the current distributions of ESA for *An. dirus* were largely affected by the annual temperature range (bio7) and precipitation of wettest quarter (bio16). The mean temperature of coldest quarter (bio11) and annual temperature

range (bio7) largely controlled the spatial distribution of ESA for *An. minimus*. The current distribution of ESA for *An. lesteri* and *An. sinensis* were both largely affected by the precipitation of driest quarter (bio17) and fraction of urban area within grid cell (gurbn).

Relationship between vector occurrence and environmental variables

A negative near-linear relationship is observed between the mean temperature annual range (bio7) and the probability of *An. dirus* presence, peaking at 20 ℃. In contrast, the precipitation of the wettest quarter (bio16) seems to be restrictive and a relative high probability of *An. dirus* presence is noted where precipitation is between 900 mm to 1,000 mm (Supplementary Fig. S3-1). Above 5mm, the precipitation of the driest quarter was negatively related to predicted probability of occurrence of *An. minimus*, while the mean temperature of the coldest quarter was positively related to predicted probability of occurrence (Supplementary Fig. S3-2). The predicted probability of occurrence for *An. lesteri* was positively related to precipitation in the wettest quarter (bio16) from 0mm to 50mm, while there was a negative relationship when greater than 50mm (Supplementary Fig. S3-3). The response curve of *An. sinensis* indicated that both the precipitation of the driest quarter (bio17) and the minimum temperature of the coldest month (bio6) were positively related to probability of occurrence (Supplementary Fig. S3-4). As our model predicted, the probability of *An. diru*s occurrence was non-linear negatively related to gurban. While predicted probability of occurrence of *An. minimus* was negatively related to gurban where gurban is below 0.03. However, both the probability of *An. lesteri* and *An. sinensis* was non-linear positively related to gurban.

Consistence among different GCM models

Generally, the simulated ESA in the same time slice agree very well among different GCMs for three malaria vector species (*An. dirus*, *An. minimus*, and *An. sinensis*), although this was not the case for *An. lesteri*. While different RCP scenarios made little difference to predictions of *An. dirus*, varying the GCMs used led to substantial differences in predicted distributions. The predicted ESA of *An. dirus* from CCCma_CanESM2 was clearly distributed in different areas, with respect to BCC-CSM1-1 and CSIRO-Mk3.6.0 based on RCP2.6, RCP4.5 and RCP8.5 in the 2030s and 2050s. However, little difference was observed in the predicted ESA

distribution for *An. minimus* under the three GCM models and three RCP scenarios in the same time slice. However, the results from all three GCMs indicated the predicted ESA for *An. lesteri* under climate change in the 2030s and 2050s changed greatly among GCMs. Most of the predictions for *An. sinensis* using the three GCMs are in agreement (Supplementary Fig. S4-S12). In general, the ESA for *An. sinensis* estimated by CCCma_CanESM2 were larger than the other two GCMs.

Assumptions, limitations, and evaluation of point sampling approach

The point sampling approach is a useful method to predict finer-resolution species distributions when only coarse presence data (e.g. county level vector presence data) are available [121]. In our study, point sampling involved assigning the presence data to a random location (random point) within each county. The set of random points were then associated with fine resolution environmental variables via an ecological niche model (e.g., Maxent) [122]. However, the approach inherits several inevitable assumptions and limitations. This approach assumes all areas are suitable for the modelled species within the county boundary [123]. This assumption may not be true due to fine scale heterogeneities in suitable habitats. As a degree of uncertainty arises in predictions derived from different random sampling iterations [124], point sampling was repeated 100 times with the mean prediction value at each fine-grain cell calculated.

In order to evaluate the uncertainty of point sampling approach, the standard deviation predictions derived from the 100 replicates was calculated (Supplementary Fig. S13) and classified into four levels: 0.00-0.05, 0.06-0.10, 0.11-0.15 and 0.16-0.28. The Supplementary Fig.S13 indicates that around 80% of the grid cell predictions had a standard deviation of less than 0.05, with a slight difference across multiple malaria vector species. This result suggests that the point sampling approach introduced relatively little uncertainty in the modeling process.

Supplementary References

1 Chen, G., Li, H. & Lin, Y. Horizontal survey on the epidemiological characteristics of malaria in Laiza city of the second special administrative region of Kachin State of Myanmar, a China-Myanmar border area. *Chin. J. Vector Biol. & Control* **23**, 352-356 (2012).

2 Chen, W., Wu, K. & Lin, M. Great achievements of anti-malaria for a half century and the present technical problems in Hainan Island. *Chin. Trop. Med.* **7**, 1994,2013-2016 (2007).

3 Liao, Z., Han, Y. & Chen, L. Observation on seasonal fluctuations of An.dirus and An.minimus and malaria infection in Wangxia township of Changjiang County, Hainan. *Chin. Trop. Med.* **10**, 1481-1482 (2010).

4 Wang, G. & Wang, S. Relationship between falciparummal ariaincidence and the number of Anopheles dirus in Hainan from 2001 to 2008. *Chin. J. Schisto. Control* **23**, 74-75 (2011).

5 Xu, D., Zhang, Y. & Liu, J. Analysis of measures in surveilance of malaira and control results in Nongken system of Hainan Province. *Chin. Trop. Med.* **7**, 220-222 (2007).

6 Bai, M., Li, Z. & Zhao, H. Analysis of the Effect on Malaria Control in Mojiang County from 2001 to 2007. *Prev. Med. Trib.* **15**, 436-438 (2009).

7 Chen, G., Xu, S. & Chen, Z. Malaria Situation of Immigrants and Local Residents in Menglian County, Yunnan Province. *Parasit. & Infect. Dis.* **3**, 71-72 (2005).

8 Chen, G. *et al.* Surveillance report on malaria epidemic and control situation in Yunnan Province in 2005. *J. of Pathog. Biol.* **3**, 144-146 (2008).

9 Fan, B., Huang, Z. & Li, L. The relation between the variation of population number of Anopheles minimus and malaria prevalence in Ailao Mountain of Yuxi city. *Chin. Trop. Med.* **5**, 45-46 (2005).

10 Fan, J., Li, H. & Zhu, J. Evaluation of measures in malaria control in Xishuangbanna Prefecture from 2002-2006. *Chin. Trop. Med.* **7**, 2289-2290 (2007).

11 Gui, S. & Chen, S. Analysis the Malaria Surveillance results in Sanming city from 2001 to 2008. *Strait J. Prev. Med.* **15**, 46-47 (2009).

12 Jiang, Z., Wu, G., Ding, L. & Wang, L. Investigate the Epidemic Situation and Prevention Strategies on the Major Vector-borne Disease. *Chin. J. Hyg. Insect. & Equip.* **14**, 440-444 (2008).

13 Li, F. Surveillance of malaria and control strategy in the last ten years in Jiangmen City. *Chin. Trop. Med.* **6**, 594-596 (2006).

14 Li, J., Qin, Y. & Du, J. Control measures and study on malaria spreading in hydropower station in Baise. *Chin. Trop. Med.* **6**, 1767-1767,1777 (2006).

15 Li, J., Tan, Y., Du, J. & Lin, Y. Anopheles species composition and activity patterns in Wangdian area, Guangxi Province. *Applied Prev. Med.* **12**, 345-347 (2006).

16 Li, T. & Wu, Z. Analysis of results of malaria control measures and effectiveness in Shidian County,Yunnan in 1951-2005. *Chin. Trop. Med.* **9**, 1117 (2009).

17 Lin, M., Wu, K. & Chen, W. Review and analysis of foucs outbreak of malaria in areas with Anopheles minimus as vector in Hainan Island. *Chin. Trop. Med.* **9**, 805-806,933 (2009).

18 Lu, Y. Human malaria surveillance results during 1996-2006 in Yulin City, Guangxi Province. *J. of Trop. Dis. & Parasitol.* **6**, 39-40 (2008).

19 Mao, W., Wu, D. & Mao, W. Investigation on Anopheles-malaria intermediary in Qianxinan state, Guizhou Province in 2007. *JPMT* **15**, 1884-1886 (2008).

20 Sheng, H. *et al.* Factors affecting malaria outbreak in Congjiang County of Guizhou Province. *Chin. J. Parasitol. Parasit. Dis.* **25**, 225-229 (2007).

21 Shi, W. *et al.* Investigation on malaria vectors in western part of China Myanmar border. *Chin. J. Parasitol. Parasit. Dis.* **29**, 134-137 (2011).

22 Zhou, S. & Tang, L. Study on new cryptic species of Anopheles minimus complex based on molecular markers with different evolution velocity. *J. of Pathog. Biol.* **1**, 416-419 (2006).

23 Wang, X., Zhao, T., Du, Z., Liu, M. & Lu, B. Study on the relationship between the

encironment changing with the house invading of Anopheles minimus. *Acta Parasitol. Med. Entomol. Sin.* **14**, 158-161 (2007).

24 Wu, N., Long, Q. & Luo, T. Analysis of the outbreak reasons of malaria for two years in Judong Village of Congjiang County in Guizhou. *Modern Prev. Med.* **35**, 3629-3631 (2008).

25 Xia, M. *et al.* Study on the mosquito community characteristics in human houses in Puwen Town, Yunnan Province. *Chin. J. Vector Bio. & Control* **17**, 458-460 (2006).

26 Zeng, L. *et al.* Resistance assay of malaria vectors to four kinds of common insecticides in some endemic areas of Hainan Province. *Chin. J. Parasitol. Parasit. Dis.* **29**, 200-203 (2011).

27 Zheng, B. *et al.* Comparative study on the resting habit of Anopheles minimus A and Anopheles minimus C in Yunnan Province. *Chin. J. Parasit. Dis. Con.* **23**, 146-149 (2005).

28 Zheng, B. *et al.* Population genetics study on Anopheles minimus in Yunnan Province by SSR-PCR. *J. of Trop. Med.* **7**, 529-532,535 (2007).

29 Zhou, H., Li, C., Wu, C., Zhang, Z. & Wang, P. Study on the mosquito community characteristics of human rooms in the lower valley of Lancang Jiang river, Yunnan province. *Chin. J. Vector Bio. & Control* **19**, 6-7 (2008).

30 Zhou, H. *et al.* Distribution of Anopheles minimus and its role in malaria transmission in the Kachin Region of Myanmar. *J. of Pathog. Biol.* **5**, 578-580,584 (2010).

31 Zhou, H. *et al.* The role of the malaria vectors in the upper valley of Mekong River in Yunnan. *Chin. J. Parasit. Dis. Con.* **18**, 407-411 (2005).

32 Zou, C. *et al.* Study on the geographical distribution of population density of Anopheles minimus and molecular identification of the species in Guangxi. *Chin. J. Vector Biol. & Control* **23**, 101-104 (2012).

33 Zou, S. Analysis the malaria surveillance results in Qingliu County. *Chin. J. Parasit. Dis. Con.* **18**, 66,70 (2005).

34 Bao, J., Zhao, L. & Wei, Y. The investigation report of mosquitoes at Meizhou Port. *Port Hyg. Control* **14**, 45-47 (2009).

35 Cao, Y. *et al.* Arboviruses isolated from mosquitoes in Liaoning province in 2008. *Chin. J. Vector Biol. & Control* **23**, 93-97 (2012).

36 Chai, P., Cheng, F., Maimaitijiang, W. & Tong, S. Analysis of the malaria epidemic situation in Xinjiang Province from 2002 to 2006. *Endemic Dis. Bull.* **22**, 59,61 (2007).

37 Cheng, S., Wang, X. & Yu, C. Analysis of the malaria epidemic situation from 2006 to 2009 in Baoying County. *Jiangsu J. Prev. Med.* **22**, 25-26 (2011).

38 Chen, S. Analysis of the malaria epidemic situation in 2000-2010 in Jiangyan City. *Seek Med. & Ask The Med.* **9**, 8 (2011).

39 Dai, Y., Deng, C. & Luo, Y. Investigation on the distribution and fluctuation of mosquitos in Xuanhan County. *Modern Prev. Med.* **37**, 156,161 (2010).

40 Duan, Z. Analysis of current conditions after basic elimination of malaria in Changning. *J. of Pathog. Biol.* **4**, 3,715 (2009).

41 Gong, D., Guo, X., Zhou, H. & Wang, P. Investigation of the prevalence of Japanese encephalitis in Jingdong County, Yunnan. *J. of Pathog. Biol.* **5**, 57-58,68 (2010).

42 Huang, Y. Investigation on mosquito in Muya Village, Baojing County. *Chin. J. Hyg. Insect. & Equip.* **14**, 427 (2008).

43 Liu, M. *et al.* Geographic information system analysis on the relationship of populations of Anopheles sinensis and An.jeyporiensis with the environment factors in Yunnan province

Chin. J. Vector Bio. & Control **19**, 275-279 (2008).
44 Lai, J. Surveillance of malaria in Taizhou municipality in Zhejiang province, 2000-2009. *Dis. Surveill.* **26**, 775-777 (2011).
45 Li, C. Investigation and analysis the seasonal fluctuation of adult mosquitoes in Meihekou City. *Chin. community psy.* **11**, 243 (2009).
46 Li, G., Fu, H., Fu, S., Shen, M. & Ding, X. Investigation of mosquitoes and mosquito-borne viruses in the Yangtze River Basin within Gansu province. *Chin. J. Vector Biol. & Control* **21**, 303-305 (2010).
47 Li, M. *et al.* Study on population dynamics of mosquitoes on Heixiazi Island, China. *Chin. J. Vector Biol. & Control* **24**, 252-253 (2013).
48 Li, Y., Sang, H., Zhao, W., Liu, Y. & Wang, P. Investigation on the distribution of mosquito species on China-Laos border port in Mengla County, Yunnan Province. *J. Med. Pest Control* **26**, 729-730 (2010).
49 Lin, C. Analysis of the malaria epidemic situation in 2006-2010 in Huanyan District. *Zhejiang Prev. Med.* **23**, 35-36 (2011).
50 Lin, K. *et al.* Analysis of epidemic situation of malaria at national malaria monitoring points in Guangxi Zhuang Autonomous Region, China in 2010. *Chin. J. Vector Biol. & Control* **24**, 317-319 (2013).
51 Liu, H. *et al.* Habits and insecticide resistance of Anopheles sinensis in Shandong province, China, 2008-2011. *Chin. J. Vector Biol. & Control* **24**, 17-18,23 (2013).
52 Liu, P. & Wang, Z. Investigation on mosquitoes population and seasonal fluctuation in Ankang City. *Chin. J. Vector Bio. & Control* **19**, 578-580 (2008).
53 Liu, P. *et al.* The investigation of the seasonal fluctuation of vector in Ankang city. *Chin. J. Vector Biol. & Control* **21**, 596-598 (2010).
54 Liu, P. *et al.* Investigation on the mosquitoes in the center urban area of Ankang City in 2010 and 2012. *J. Med. Pest Control* **29**, 856-858 (2013).
55 Liu, W., Yang, S. & Tang, J. Monitoring and analysis the Anopheles population and density in Funing County from 2010 to 2011. *Jiangsu J. Prev. Med.* **24**, 43-44 (2013).
56 Liu, X., Su, C. & Zhang, X. Analysis of the mosqutios distribution and the situation of mosquito-borne disease in Changchun. *Chin. J. Vector Bio. & Control* **17**, 310 (2006).
57 Liu, Y., Chen, J. & Chen, W. Study of the insecticide sensitivity of Anopheles sinensis in areas of Henan Province. *J. of Pathog. Biol.* **8**, 250,265-267 (2013).
58 Liu, Z. *et al.* Surveillance on vector mosquitoes of Japanese encephalitis in Kaijiang county, Sichuan province in 2011. *Chin. J. Vector Biol. & Control* **23**, 356-358 (2012).
59 Lou, P., Yu, J. & Zhang, L. Discussion on the malaria endemic features and monitor objects in the later stage of eliminating malaria in Pizhou City. *China Modern Doctor* **46**, 116-117 (2008).
60 Lu, W., Huang, Y. & Duan, L. Analysis on correlation between mosquito-borne diseases and seasonal fluctuation of mosquito density in Hunan Province from 2007 to 2011. *Practical Prev. Med.* **19**, 1662-1664 (2012).
61 Lu, Z., Ding, J., Fu, S., Gao, X. & Liang, G. Isolation and identification of arboviruses in Liaoning province , 2009. *Dis. Surveill.* **26**, 179-181,192 (2011).
62 Ouyang, R., Xie, H., Yang, F. & Lin, Y. Malaria surveillance analysis during 2006-2012 in Fujian Province. *Strait J. Prev. Med.* **19**, 73-74 (2013).

63 Shen, Y. *et al.* Effects of different land use types on mosquito community. *Chin. J. of Ecol.* **31**, 1751-1755 (2012).
64 Wan, X. Ecology investigation of anopheles sinensis at Yellow River delta. *J. Med. Pest Control* **28**, 794-795 (2012).
65 Wang, B. & Zhang, B. The monitoring and analysis of seasonal fluctuation of the Main four vectors in Luxian county during 2007. *Med. Ani. Prev.* **24**, 898-900 (2008).
66 Wang, F. *et al.* Investigation of mosquito species from three provinces of Northeast China. *Chin. J. Vector Bio. & Control* **17**, 476-480 (2006).
67 Wang, H., Wang, H. & Cheng, P. Survey of mosquitoes in Hanzhuang malaria monitoring site in Shandong Province. *Chin. Trop. Med.* **9**, 46-48 (2009).
68 Wang, P., Zhou, H. & Wu, C. Investigation of mosquito species,distributions and larva breeding habits in the low treaches of Lancangjiang River in Yunnan. *Chin. Trop. Med.* **11**, 657-659 (2011).
69 Wang, W., Cao, J. & Zhou, H. Seasonal increase and decrease of malaria vector in monitoring sites of Jiangsu Province in 2005-2010. *Chin. Trop. Med.* **13**, 152-155 (2013).
70 Wang, W., Zhou, H. & Liu, Y. Comparison of seasonal fluctuation and nocturnal activity patterns of Anopheles sinensis in different regions of Jiangsu pronince. *Chin. Trop. Med.* **13**, 292-295 (2013).
71 Wang, X. Observation on the parous rate of Anopheles sinensis in different time period at night in Zhangjiagang city. *Chin. Trop. Med.* **13**, 367-368 (2013).
72 Wu, W., Zhou, Y., Pang, W., Song, L. & Qi, S. Investigation on mosquito population and seasonal fluctuation in Dalian city. *Chin. J. Hyg. Insect. & Equip.* **18**, 154-155 (2012).
73 Xu, C. Epidemiological survey of malaria in Shangqiu City in 2000-2010. *Modern Prev. Med.* **38**, 5516-5516，5521 (2012).
74 Xu, T. *et al.* Changes of pyrethroid resistance and P450 monooxygenase activity with age in Anopheles sinensis in Huainan City, Anhui Province,China. *Chin. J. Schisto. Control* **25**, 157-159,166 (2013).
75 Yang, G., Shao, M. & Yu, H. Threshold of anopheles in malaria transmission in Shuangliu County. *Parasit. & Infect. Dis.* **11**, 26-28 (2013).
76 Yang, T., Wang, J. & Wang, X. Investigation of mosquito species and mosquito-borne arboviruses in Haikou, China. *Chin. J. Vector Biol. & Control* **24**, 254-256 (2013).
77 Yang, Z., Luo, D. & Zhang, L. Analysis of the characteristics of malaria epidemics in Hongya County. *J. of Pathog. Biol.* **4**, 2,593 (2009).
78 Yu, T., Sun, X. & Wang, G. Analysis on monitoring data on mosquito density at longkou monitoring point,Yangtai City,2009-2011. *Prev. Med. Trib.* **19**, 358-359 (2013).
79 Zhang, J. Surveillance results on malaria in Zhoushan Island from 2006 to 2011. *Zhejiang Prev. Med.* **24**, 41-43 (2012).
80 Zhang, J. & Gong, Z. General situation on mosquito research in China. *Chin. J. Vector Bio. & Control* **19**, 595-599 (2008).
81 Zhang, P., Chen, J. & Gao, Y. Monitor result of in malaria area in Dandong city. *Chin. J. Hyg. Insect. & Equip.* **18**, 229-231 (2012).
82 Zhang, X., Zhou, C., He, Y. & Zhang, L. Analysis of the surveillance results of a national monitoring site from 2005 to 2010 in Qingshen county,Sichuan Province *Journal of Occupational Health and Damage* **26**, 386-388 (2011).

83 Zhang, Y., Wang, J. & Ying, Y. Monitoring on the populations of mosquitoes and seasonal fluctuation in Xianju County. *Zhejiang Prev. Med.* **19**, 36 (2007).

84 Zhang, Y., Liu, J. & Guo, X. Resistance of Anopheles sinensis to Insecticides in Huaibin County of Henan Province. *Chin. J. Parasitol. Parasit. Dis.* **30**, 493-495 (2012).

85 Zhao, X. *et al.* Changes in species composition and densities of mosquitoes in Three Gorges Reservoir Area in Yichang, Hubei province, China during 1997-2012. *Chin. J. Vector Biol. & Control* **24**, 425-428 (2013).

86 Zheng, X., Tan, G. & Shu, Y. Vector Investigation in Beichuan county of Sichuan earthquake-stricken areas in 2009. *Chin. J. Parasit. Dis. Con.* **22**, 606-610 (2011).

87 Zhou, M. & Zhang, J. Analysis of the surveillance results on malaria in district from 1998 to 2007. *Zhejiang Prev. Med.* **21**, 24-25 (2009).

88 Zhu, H., Zhang, L., Yang, S., Jin, D. & Feng, Y. Analysis of the surveillance results of malaria intermediary in Yongsheng County,Yunnan Province. *Soft Science of Health* **26**, 456-458 (2012).

89 Chen, G., Li, W. & Ren, Z. Serum epidemiological surveillance of malaria distribution of Anopheles anthropophagus in Yunnan Province. *Parasit. & Infect. Dis.* **4**, 130-131 (2006).

90 Chen, X., Hen, X., Shen, D. & Liu, S. Evaluation of prevention effect of falciparum malaria in Xinyang City, Henan Province. *Chin. J. Parasit. Dis. Con.* **18**, 319,320 (2005).

91 Chuan, K., LI, J. & Qin, Y. Investigation on geographical distribution, ecological feature and malaria transmission of Anopheles in Guangxi. *Chin. J. Vector Bio. & Control* **18**, 112-115 (2007).

92 Guang, Q., Zhang, H. & Li, H. Evaluate the effectiveness of measures for mosquito control in Anopheles anthropophagus area in Hubei Province. *Chin. J. Vector Biol. & Control* **16**, 261-263 (2005).

93 Gui, S. & Chen, S. Feasibility analysis of the elimination of malaria in Sanming. *Strait J. Prev. Med.* **18** (2012).

94 Guo, C. Advance in the research of Anopheles anthropophagus in China. *Chin. Trop. Med.* **5**, 384,396-398 (2005).

95 Kang, Y., Xu, G. & Yang, W. Egg and adult morphological characters of Anopheles anthropophagus from five different regions of China. *Chin. J. Vector Bio. & Control* **16**, 339-341 (2005).

96 Lei, Y., Xu, G. & Xiao, N. Surveillance of malaria in Sichuan Province, 2006-2008. *J. Prev. Med. Inf.* **26**, 107-110 (2010).

97 Li, H. & Chen, G. Study on malaria control strategies in the malaria epidemic areas transmitted by Anopheles anthropophagus in Yunnan province. *Chin. J. Vector Bio. & Control* **20**, 569-572 (2009).

98 Li, H., Chen, G. & Long, X. Implementation of malaria control measures in sporadic outbreak and resurgence in Yunnan Province and appraisal of results. *Chin. Trop. Med.* **8**, 601-603 (2008).

99 Li, J., Zhou, H., Jin, X. & Zhu, G. Observation on development time of Anopheles anthropophagus in different areas of China. *Chin. J. Schisto. Control* **18**, 390-391 (2006).

100 Li, L., Xu, G. & Xiao, N. Analysis on the malaria surveillance in supervised counties in Sichuan Procince from 2006 to 2009. *Modern Prev. Med.* **38**, 1530-1532,1534 (2011).

101 Li, W., Liu, F., Zhang, Q. & Dong, J. Analysis of the monitoring data of the density of

mosquitoes in 2008~2009 in Longgang District,Shenzhen Province. *South China J. Prev. Med.* **36**, 69-70 (2010).
102 Lin, W. & Mao, Z. Analysis of malaria epidemic in Hubei Province (2009-2011). *J. of Pub. Health and Prev.* **23**, 15-17 (2012).
103 Liu, S., Zhang, R. & Yan, J. Research on malaria control strategies of Anopheles anthropophagus distribution areas in Yibin. *J. Prev. Med. Inf.* **22**, 205-209 (2006).
104 Meng, R. *et al.* Evaluation effective of malaria control in huanjiang maonan Nationality Autonomous County in Guangxi in 1957-2007. *J. of Trop. Med.* **9**, 1306-1309 (2009).
105 Ouyang, R., Xie, H., Yang, F. & Lin, Y. Analysis of the malaria surveillance data in Fujian Province from 2006 to 2012. *Strait J. Prev. Med.* **19**, 73-74 (2013).
106 Pan, B. *et al.* Compare the capabilities of malaria transmission between Anopheles anthropophagus and Anopheles sinensis in Guangdong Province. *J. of Trop. Med.* **8**, 1169-1171 (2008).
107 Pan, B. *et al.* Relationship between the distribution of Anopheles anthropophagus and the conditions of natural geography and social economy in Guangdong province. *Chin. J. Zoonoses* **21**, 62-65,69 (2005).
108 Pan, J. *et al.* Impact of pesticide use on the density of Anopheles anthropophagus and malaria incidence. *Chin. J. Parasitol. Parasit. Dis.* **30**, 218-223 (2012).
109 Lin, L. *et al.* Development and Planning of Vector control discipline in Fujian Province. *Hai Xia Ke Xue* **1**, 119-128 (2013).
110 Qu, F. Discussion on the status of Anopheles anthropophagus and Anopheles lesteri in China. *Chin. J. Parasitol. Parasit. Dis.* **23**, 243-245 (2005).
111 Shang, L. *et al.* Study on distribution, ecological feature and malaria transmission effect of Anopheles anthropophagus in Henan Province, China. *J. of Pathog. Biol.* **2**, 304-306 (2007).
112 Shao, Y., Fan, Z. & Li, C. Prevalent features of malaria in Xiangzhou District of Xiangyang City in 2007-2011. *Chin. Trop. Med.* **12**, 1077-1078,1081 (2012).
113 Wang, W. *et al.* Survey on malaria vectors in Jiangsu Province, 2005-2009. *Chin. J. Schisto. Control* **23**, 453-456 (2011).
114 Xu, B. *et al.* Evaluation and surveillance on the effect of control for the Anopheles anthropophagus in Fujian province. *Chin. J. Zoonoses* **25**, 110-114 (2009).
115 Xu, B., Su, Y. & Liu, Y. Malaria evaluation on the control effect in Henan Province during 1970-2010. *Henan J. Prev. Med.* **22**, 321-326 (2011).
116 Ye, D. *et al.* Research on the remnants Anopheles anthropophagus and the role of malaria transmission in Fuzhou. *Med. Ani. Prv.* **22**, 549-550 (2006).
117 Yu, Y. Anopheles anthropophagus-the species of mosquito transmit malaria in China. *China Nature* **4**, 61 (2007).
118 Zhang, S., Guo, C. & Guo, L. Epidemiological analysis of malaria situation in Xiayi County of Henan Province in 2003-2009. *Chin. Trop. Med.* **11**, 830-831 (2011).
119 Zheng, X. *et al.* Morphology and habits of An. anthropophagus and its role in malaria transmission in Hengqin Island of Zhuhai City. *Chin. J. Parasitol. Parasit. Dis.* **25**, 488-491 (2007).
120 Zhou, H. *et al.* Susceptibility of Anopheles anthropophagus from different areas of China to Plasmodium vivax. *Chin. J. Schisto. Control* **20**, 265-268 (2008).
121 McPherson, J. M., Jetz, W. & Rogers, D. J. Using coarse-grained occurrence data to predict

species distributions at finer spatial resolutions—possibilities and limitations. *Ecol. Model.* **192**, 499-522 (2006).

122 Niamir, A., Skidmore, A. K., Toxopeus, A. G., Muñoz, A. R. & Real, R. Finessing atlas data for species distribution models. *Divers. Distrib.* **17**, 1173-1185 (2011).

123 Keil, P., Belmaker, J., Wilson, A. M., Unitt, P. & Jetz, W. Downscaling of species distribution models: a hierarchical approach. *Methods Ecol. Evol.* **4**, 82-94 (2013).

124 Costa, G., Nogueira, C., Machado, R. & Colli, G. Sampling bias and the use of ecological niche modeling in conservation planning: a field evaluation in a biodiversity hotspot. *Biodivers. Conserv.* **19**, 883-899 (2010).

Supplementary Table S1. Search results of presence for the four malaria vectors.

Species	References
An.dirus (AD)	1-5
An.minimus (AM)	2, 3, 5-33
An.lesteri (AL)	20, 24, 89-120
An.sinensis (AS)	34-88

Supplementary Table S2. Bioclimatic and land use variables used in the final model for the four anopheles species.

Variable Code	Species	Name	Unit
bio2	AL, AS	Mean Diurnal Range	℃
bio6	AL, AS	Min Temperature of Coldest Month	℃
bio7	AD, AM	Temperature Annual Range	
bio10	AL, AS	Mean Temperature of Warmest Quarter	℃
bio11	AD, AM	Mean Temperature of Coldest Quarter	℃
bio16	AM, AL, AS	Precipitation of Wettest Quarter	mm
bio17	AM, AL, AS	Precipitation of Driest Quarter	mm
gcrop	AD, AM, AL, AS	Fraction of each grid cell in cropland	%
gothr	AD, AM, AL, AS	Fraction of each grid cell in primary vegetation land	%
gpast	AD, AM, AL, AS	Fraction of each grid cell in pasture	%
gsecd	AD, AM, AL, AS	Fraction of each grid cell in secondary vegetation land	%
gurbn	AD, AM, AL, AS	Fraction of each grid cell in urban land	%
gwater	AD, AM, AL, AS	Fraction of each grid cell in water area	%

Supplementary Table S3. Estimation of current and future stable, gained and lost enviromentally suitable area (ESA) (unit: thousand square kilometres) for four malaria vectors, from Maxent model using RCP2.6, RCP4.5 and RCP8.5 climate scenarios derived from an ensemble of simulations from three general circulation models (BCC-CSM1-1, CCCma_CanESM2 and CSIRO-Mk3.6.0) during the 2030s and 2050s.

Species	Current	2030s			2050s		
	ESA	Stable	Gained	Lost	Stable	Gained	Lost
An. dirus	186.8						
RCP2.6		171.9	71.9	14.9	164.3	60.2	22.6
RCP4.5		179.0	112.5	7.8	168.3	79.7	18.5
RCP8.5		173.4	88.3	13.4	168.6	89.6	18.2
An. minimus	779.0						
RCP2.6		844.8	111.4	45.5	686.1	51.0	143.8
RCP4.5		867.8	126.4	37.6	737.9	63.4	104.5
RCP8.5		884.9	145.1	39.1	799.9	102.7	81.8
An. lesteri	1613.5						
RCP2.6		1850.7	395.4	158.2	1790.5	457.1	280.1
RCP4.5		1840.9	390.3	160.0	1930.3	576.5	259.7
RCP8.5		1903.7	466.2	171.0	2125.1	718.7	207.2
An. sinensis	2690.9						
RCP2.6		2895.2	227.0	22.6	2885.9	256.0	60.9
RCP4.5		2866.0	199.8	24.6	2885.4	258.9	64.3
RCP8.5		2911.1	245.5	25.3	3012.1	377.4	56.2

Supplementary Table S4. Estimation of exposed human population (unit: millions) within stable, gained and lost ESA for four malaria vectors, from Maxent model using RCP2.6, RCP4.5 and RCP8.5 climate scenarios derived from an ensemble of simulations from three general circulation models (BCC-CSM1-1, CCCma_CanESM2 and CSIRO-Mk3.6.0) during the 2030s and 2050s periods.

Species	Current	2030s			2050s		
	Population	Stable	Gained	Lost	Stable	Gained	Lost
An. dirus	26.4						
RCP2.6		14.1	7.9	3.1	11.6	5.1	4.4
RCP4.5		15.9	12.4	1.3	12.3	8.8	3.7
RCP8.5		14.1	8.8	3.1	12.2	8.4	3.8
An. minimus	162.8						
RCP2.6		100.4	17.7	5.6	99.4	11.3	18.1
RCP4.5		101.6	19.5	4.3	105.1	15.0	12.5
RCP8.5		101.0	21.2	5.0	107.0	19.2	10.5
An. lesteri	619.0						
RCP2.6		348.5	65.4	54.0	373.3	98.0	105.0
RCP4.5		347.2	60.9	55.3	384.6	132.8	93.8
RCP8.5		350.3	88.1	52.1	405.7	162.8	72.7
An. sinensis	1005.2						
RCP2.6		649.8	33.3	3.7	792.8	44.3	9.6
RCP4.5		648.2	30.3	5.3	790.2	44.2	12.2
RCP8.5		649.3	34.6	4.2	790.8	58.9	11.5

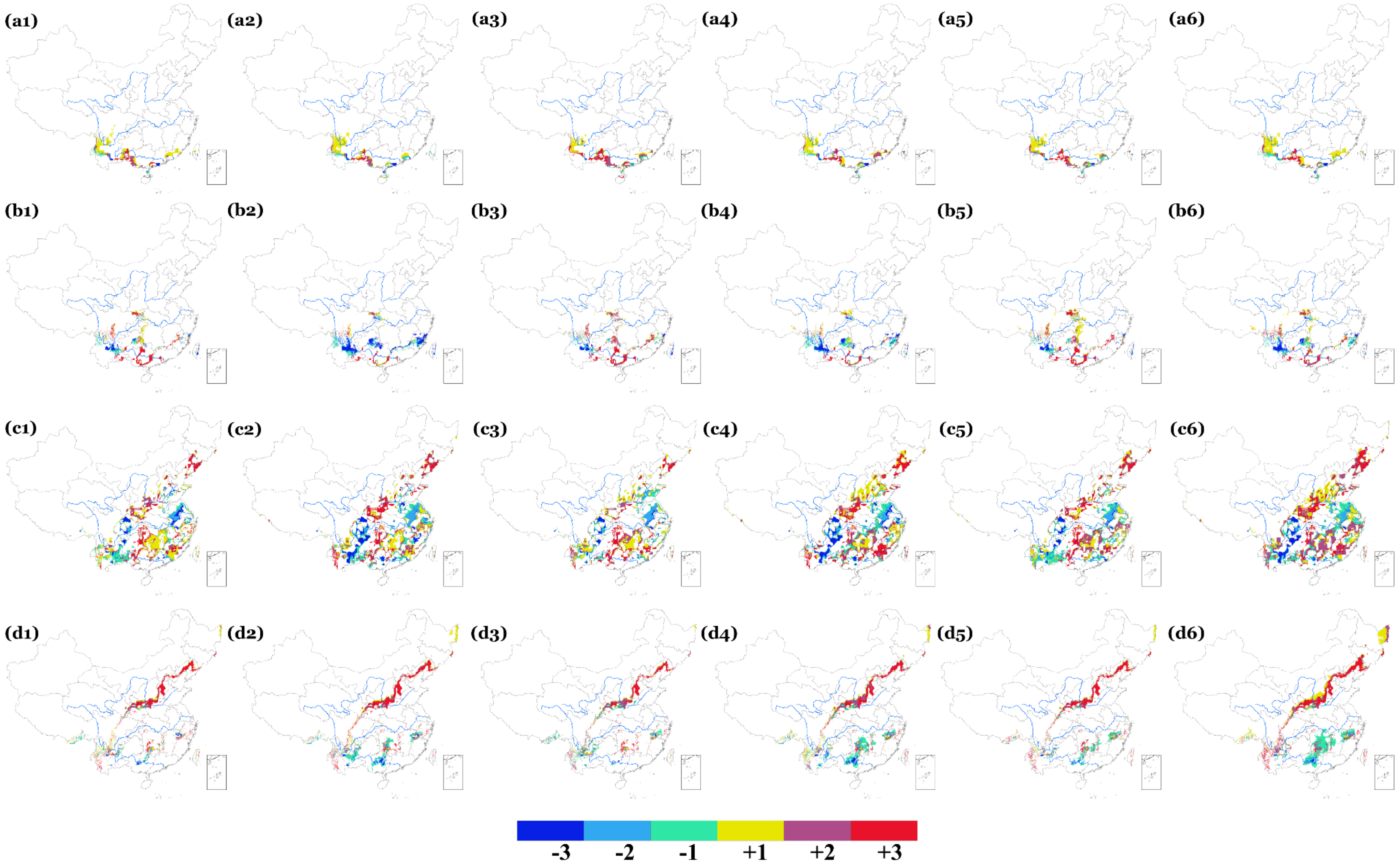
(a1)
(a2)
(a3)
(a4)
(a5)
(a6)
(b1)
(b2)
(b3)
(b4)
(b5)
(b6)
(c1)
(c2)
(c3)
(c4)
(c5)
(c6)
(d1)
(d2)
(d3)
(d4)
(d5)
(d6)
-3
-2
-1
+1
+2
+3

Supplementary Figure S1. Estimation of changes (gained and lost) in spatial distribution of four dominant malaria vectors in the 2030s and 2050s. The first two columns indicate RCP 2.6 in the 2030s and 2050s; The second two columns indicate RCP 4.5 in the 2030s and 2050s; The third two columns indicate RCP 8.5 in the 2030s and 2050s. The different colors show the number of GCMs which agree on the ESA for malaria vectors. Positive values indicate gained ESA and negative values indicate lost ESA. Maps created in ArcGIS 10.2 (Environmental Systems Resource Institute, ArcMap Release 10.2, ESRI, Redlands, California).

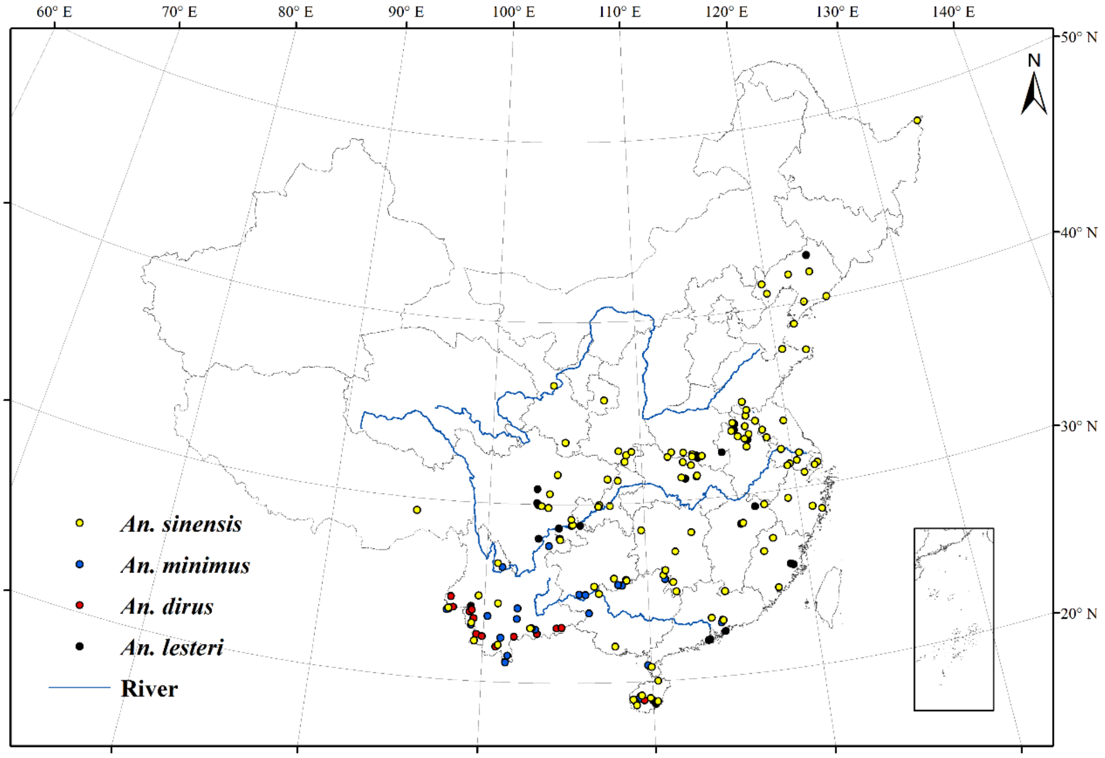

Supplementary Figure S2. The distribution of presence records (centroid of county) of four major malaria vectors in China. Maps created in ArcGIS 10.2 (Environmental Systems Resource Institute, ArcMap Release 10.2, ESRI, Redlands, California).

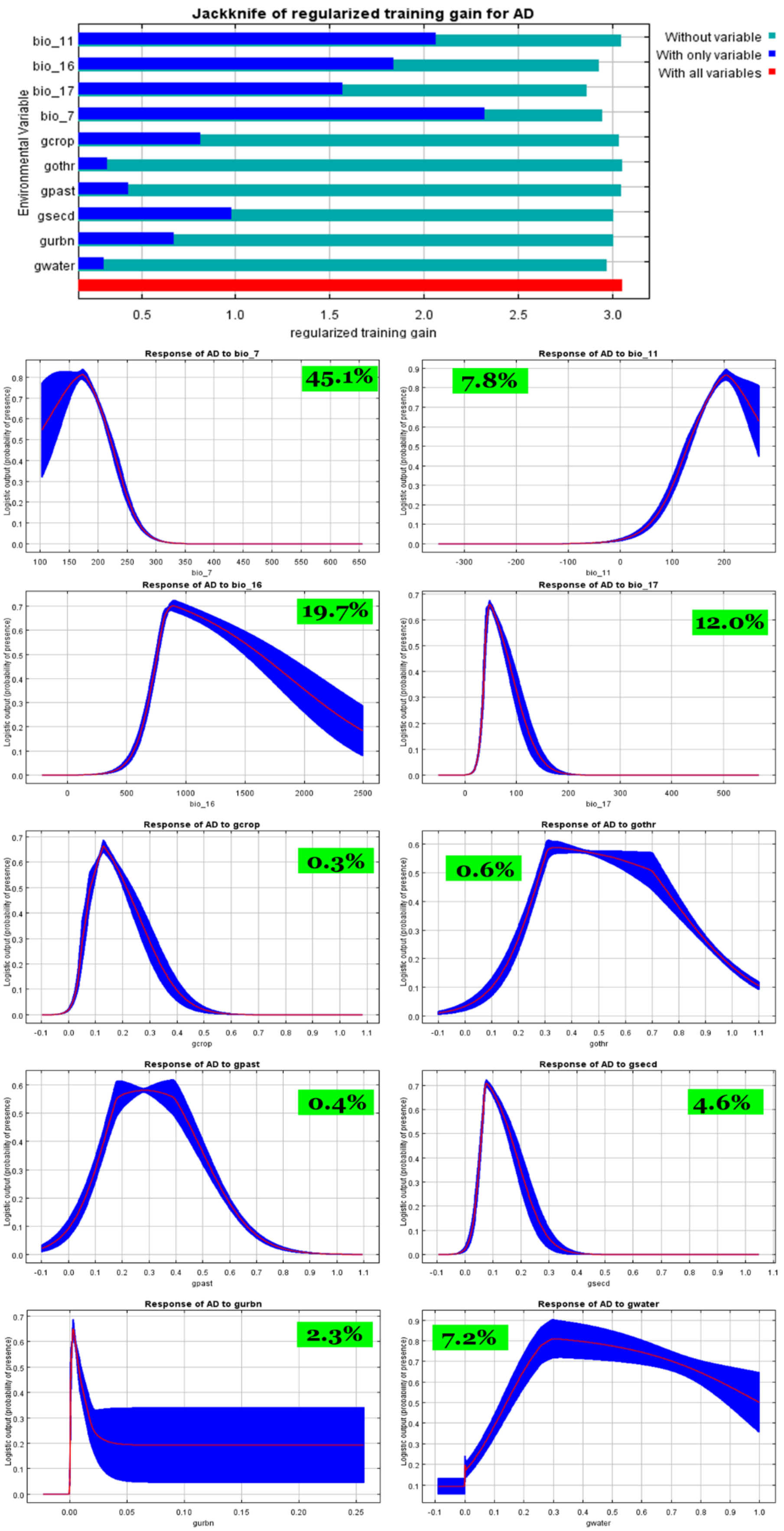

Figure S3-1 The jackknife of regularized training gain for *An.dirus*

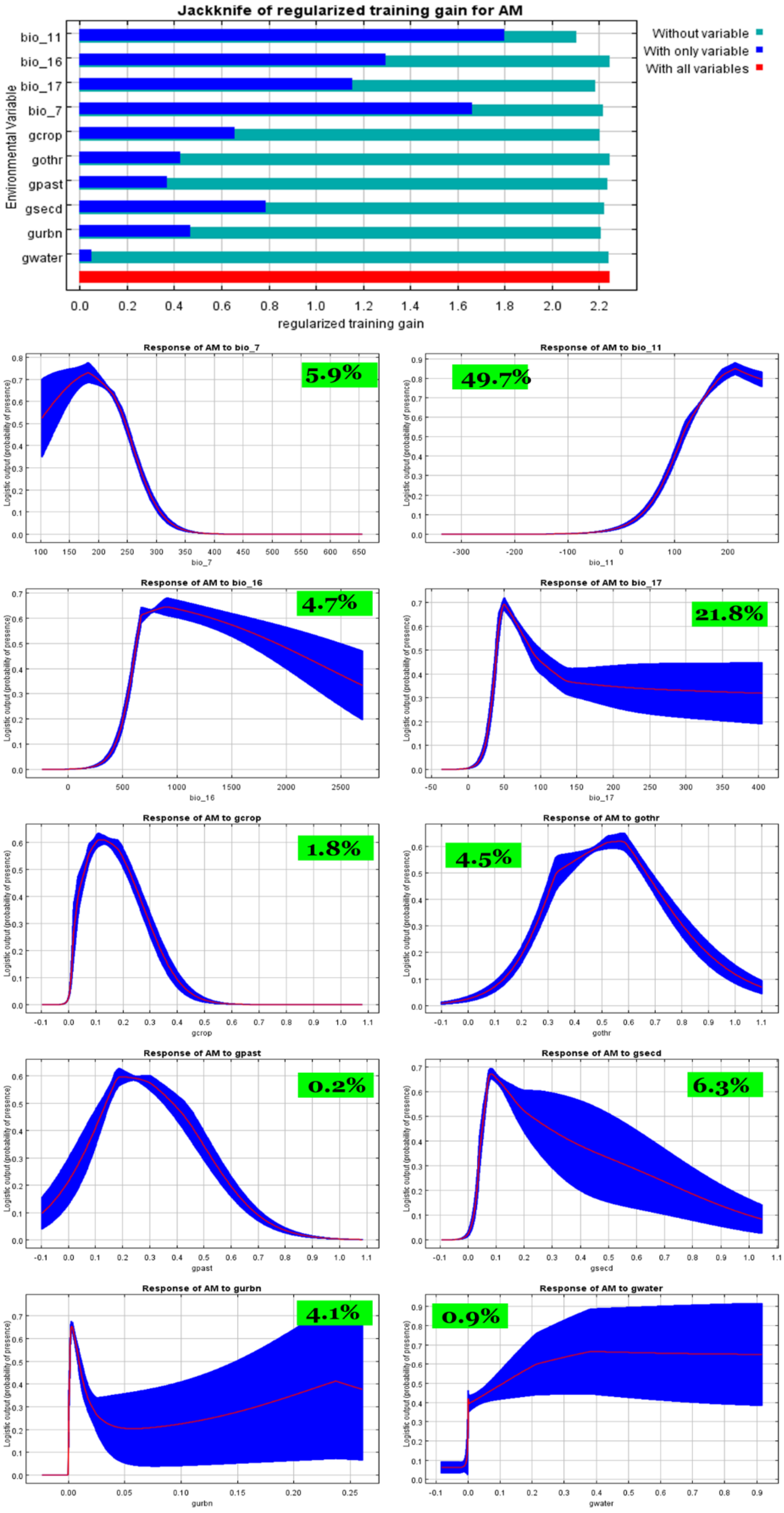

Figure S3-2 The jackknife of regularized training gain for *An.minimus*

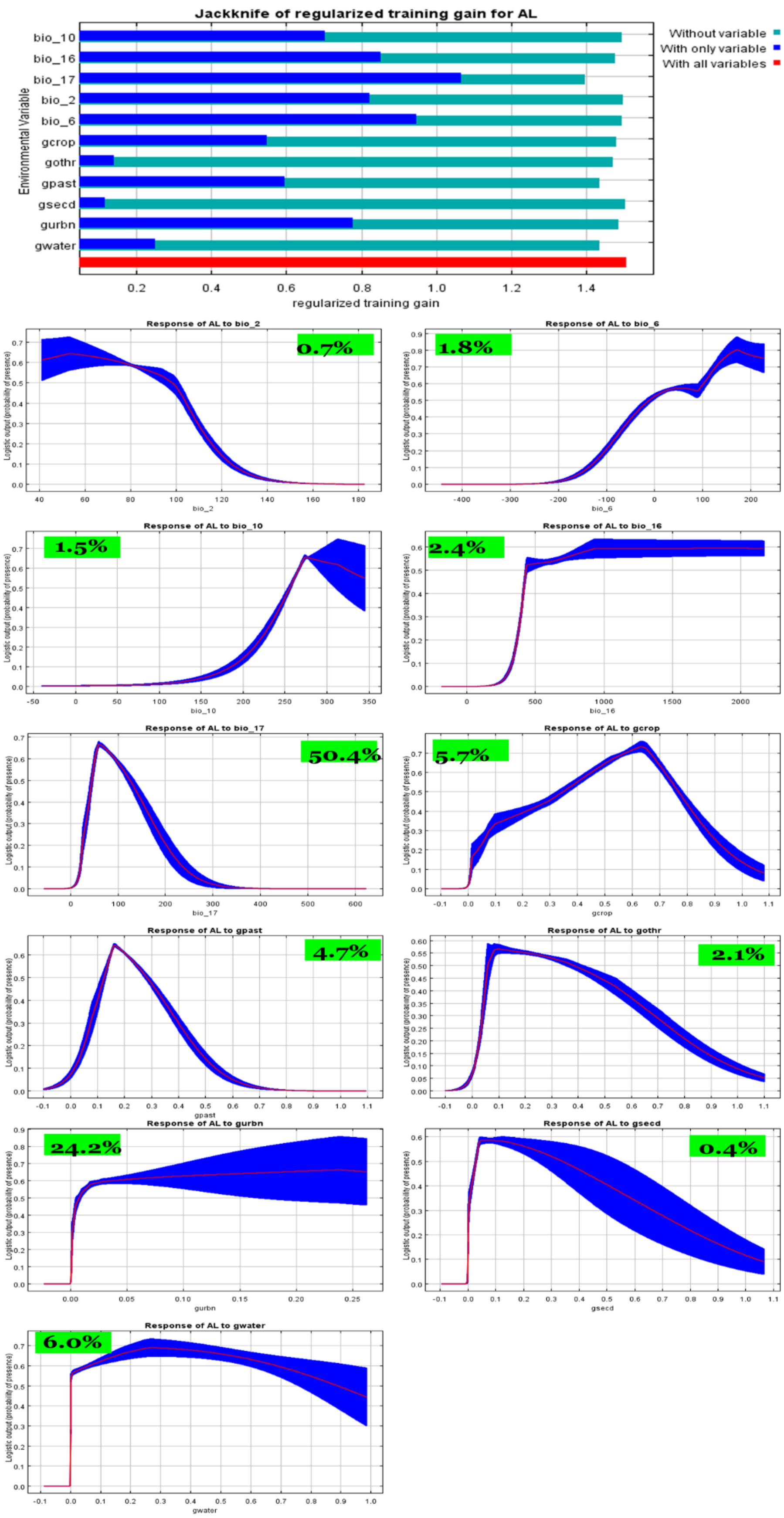

Figure S3-3 The jackknife of regularized training gain for *An. lesteri*

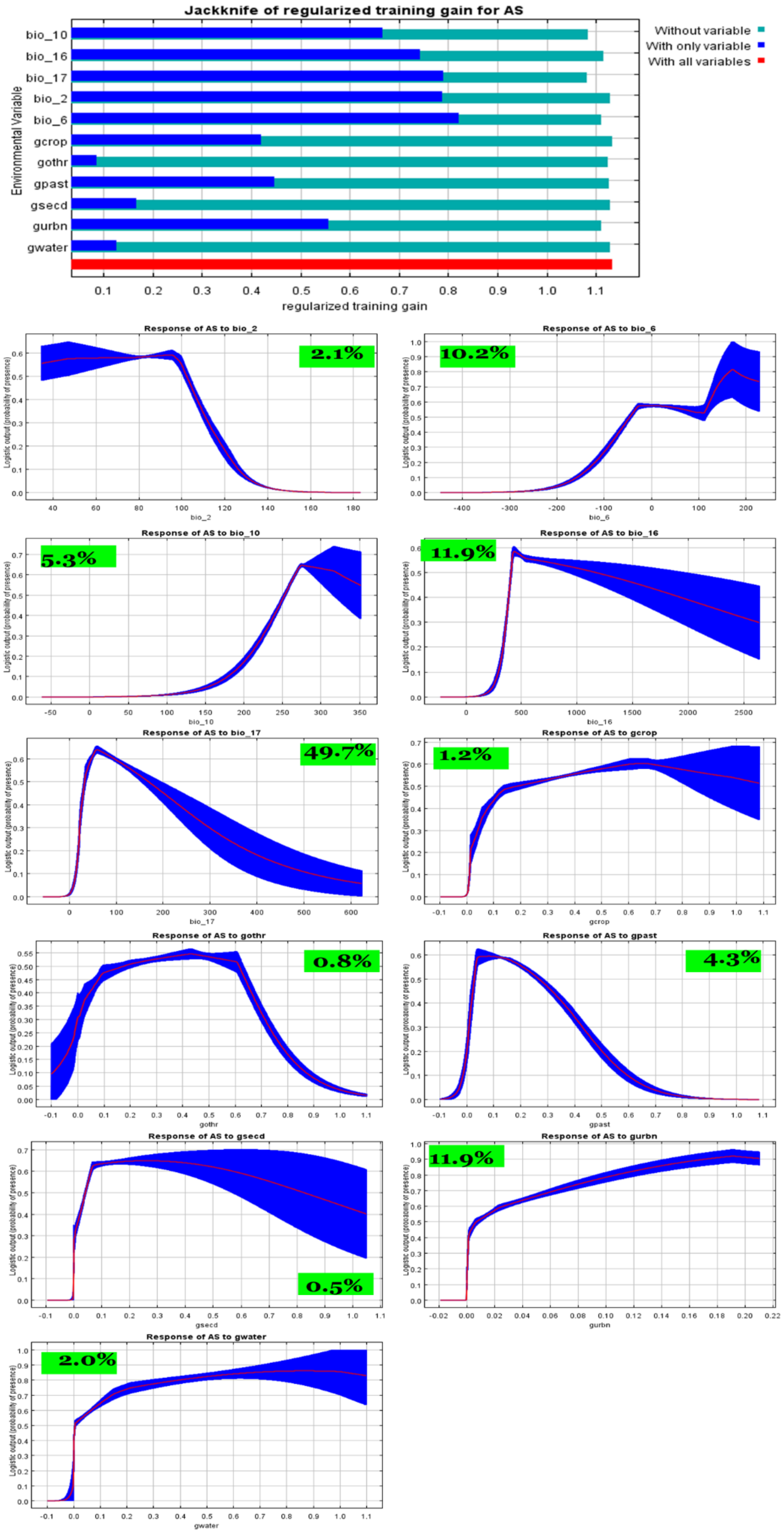

Figure S3-4 The jackknife of regularized training gain for *An. sinensis*

Supplementary Figure S3. Jacknife test and marginal response curve of four malaria vectors to six climatic variables from the Maxent model, with varaible importance on each graph. The y-axes indicate probability of presence. AD for *An. dirus*; AM for *An. minimus*; AL stand for *An.lesteri*; AS for *An. sinensis*. Response curves showing relationships between probability of presence of species and six selected bioclimatic predictors of the four malaria vectors. Values shown are average 30 replicate runs; blue margins show ±1 SD calculated 30 replicates.

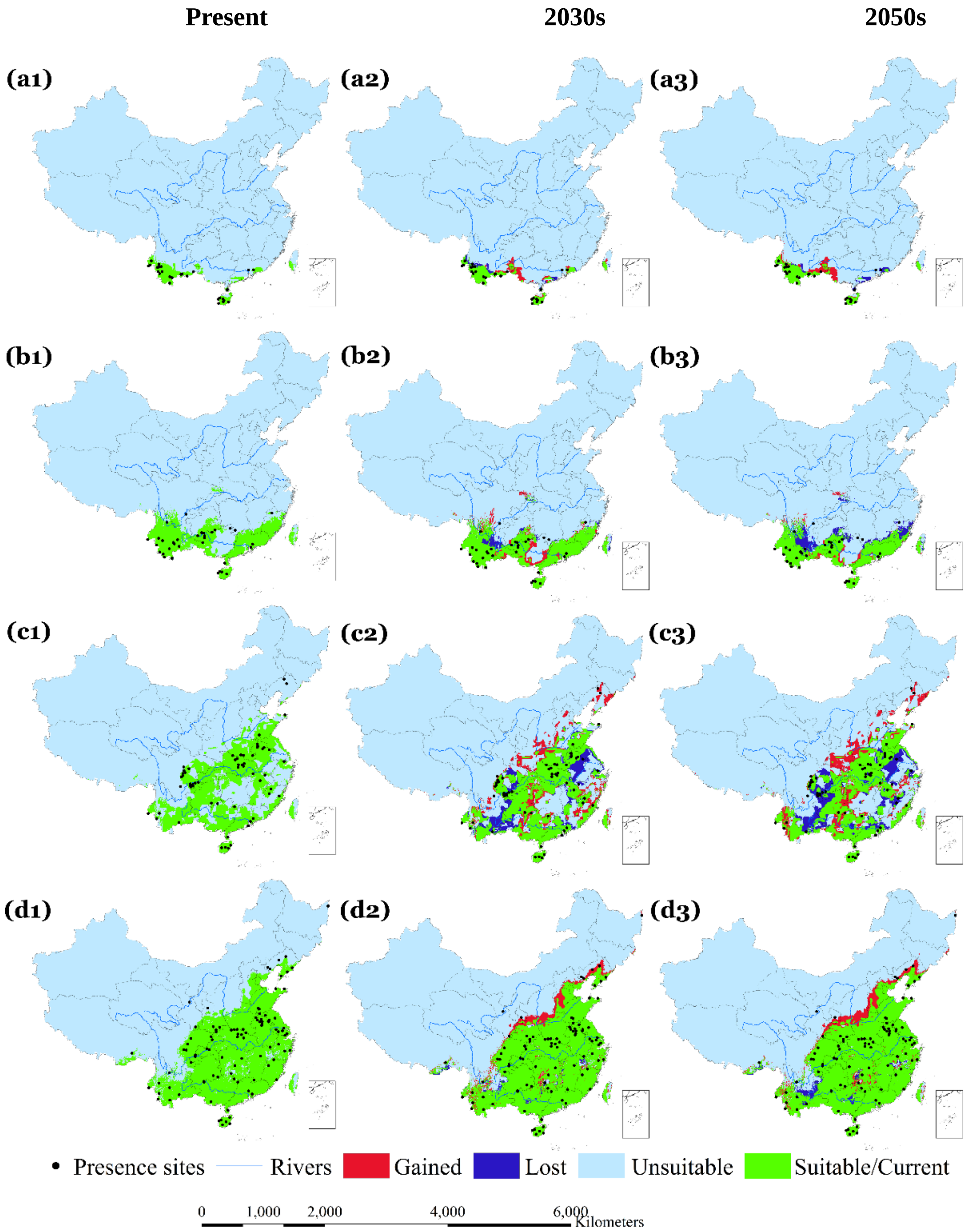

Supplementary Figure S4. Potential current (suitable and unsuitable) and future (suitable/stable, lost, gained and unsuitable) ESA for *An. dirus* (a1-a3), *An. minimus* (b1-b3), *An. lesteri* (c1-c3), *An. sinensis* (d1-d3). Future predictions are based on **BCC-CSM1-1** climate model for the 2030s and 2050s (**RCP2.6**). The second and third column indicate the 2030s and 2050s, respectively. The black dots indicate occurrence localities of four malaria vectors currently. The green color shows stable suitable areas, blue shows lost ESA and red shows gained ESA. All the lost and gained areas were calculated based on current distribution as reference. Maps created in ArcGIS 10.2 (Environmental Systems Resource Institute, ArcMap Release 10.2, ESRI, Redlands, California).

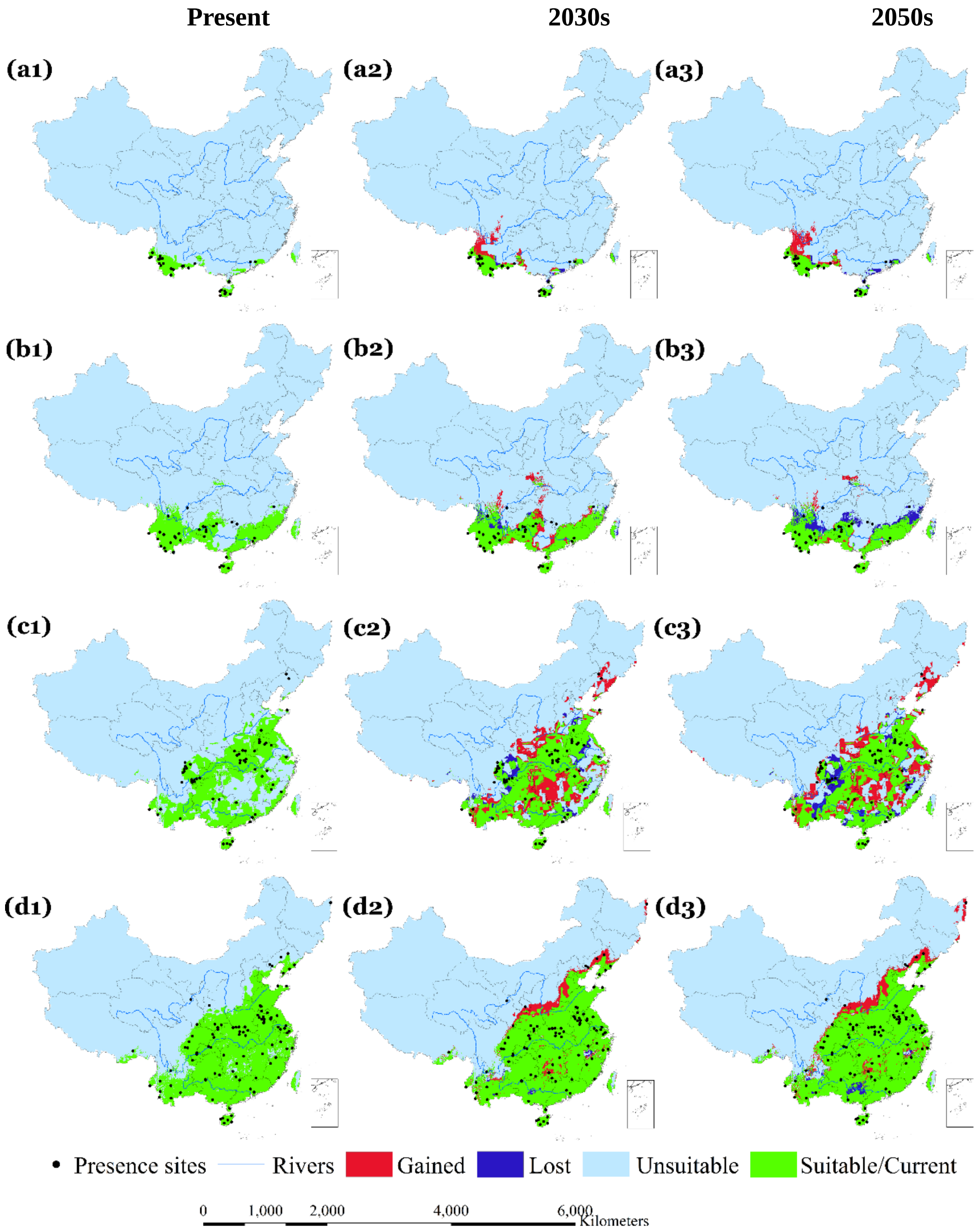

Supplementary Figure S5. Potential current (suitable and unsuitable) and future (suitable/stable, lost, gained and unsuitable) ESA for *An. dirus* (a1-a3), *An. minimus* (b1-b3), *An. lesteri* (c1-c3), *An. sinensis* (d1-d3). Future predictions are based on **CCCma_CanESM2** climate model for the 2030s and 2050s (**RCP2.6**). The second and third column indicate the 2030s and 2050s, respectively. The black dots indicate occurrence localities of four malaria vectors currently. The green color shows stable suitable areas, blue shows lost ESA and red shows gained ESA. All the lost and gained areas were calculated based on current as reference. Maps created in ArcGIS 10.2 (Environmental Systems Resource Institute, ArcMap Release 10.2, ESRI, Redlands, California).

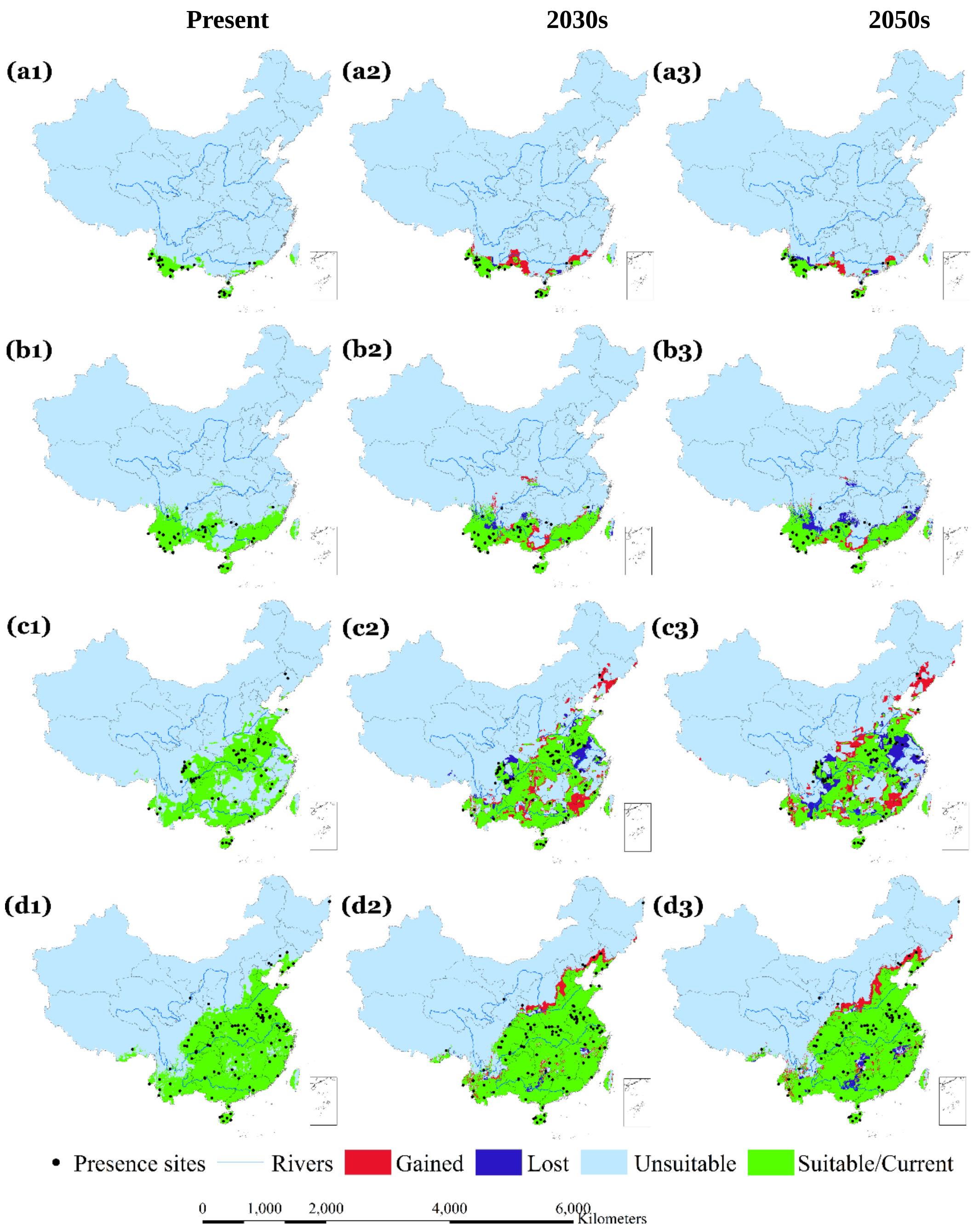

Supplementary Figure S6. Potential current (suitable and unsuitable) and future (suitable/stable, lost, gained and unsuitable) ESA for *An. dirus* (a1-a3), *An. minimus* (b1-b3), *An. lesteri* (c1-c3), *An. sinensis* (d1-d3). Future predictions are based on **CSIRO-Mk3.6.0** climate model for the 2030s and 2050s (**RCP2.6**). The second and third column indicate the 2030s and 2050s, respectively. The black dots indicate occurrence localities of four malaria vectors currently. The green color shows stable suitable areas, blue shows lost ESA and red shows gained ESA. All the lost and gained areas were calculated based on current distribution as reference. Maps created in ArcGIS 10.2 (Environmental Systems Resource Institute, ArcMap Release 10.2, ESRI, Redlands, California).

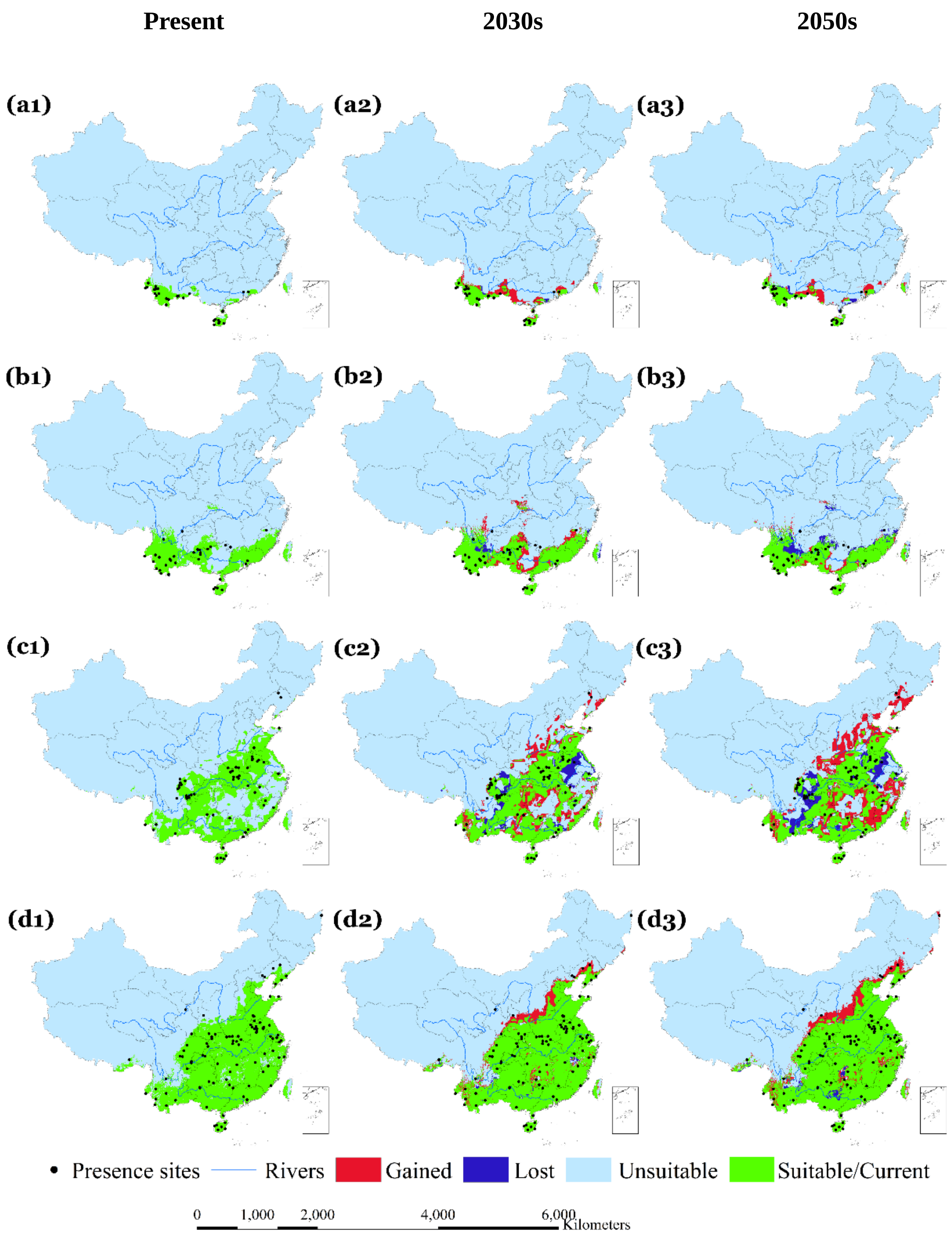

Supplementary Figure S7. Potential current (suitable and unsuitable) and future (suitable/stable, lost, gained and unsuitable) ESA for *An. dirus* (a1-a3), *An. minimus* (b1-b3), *An. lesteri* (c1-c3), *An. sinensis* (d1-d3). Future predictions are based on **BCC-CSM1-1** climate model for the 2030s and 2050s (**RCP4.5**). The second and third column indicate the 2030s and 2050s, respectively. The black dots indicate occurrence localities of four malaria vectors currently. The green color shows stable suitable areas, blue shows lost ESA and red shows gained ESA. All the lost and gained areas were calculated based on current distribution as reference. Maps created in ArcGIS 10.2 (Environmental Systems Resource Institute, ArcMap Release 10.2, ESRI, Redlands, California).

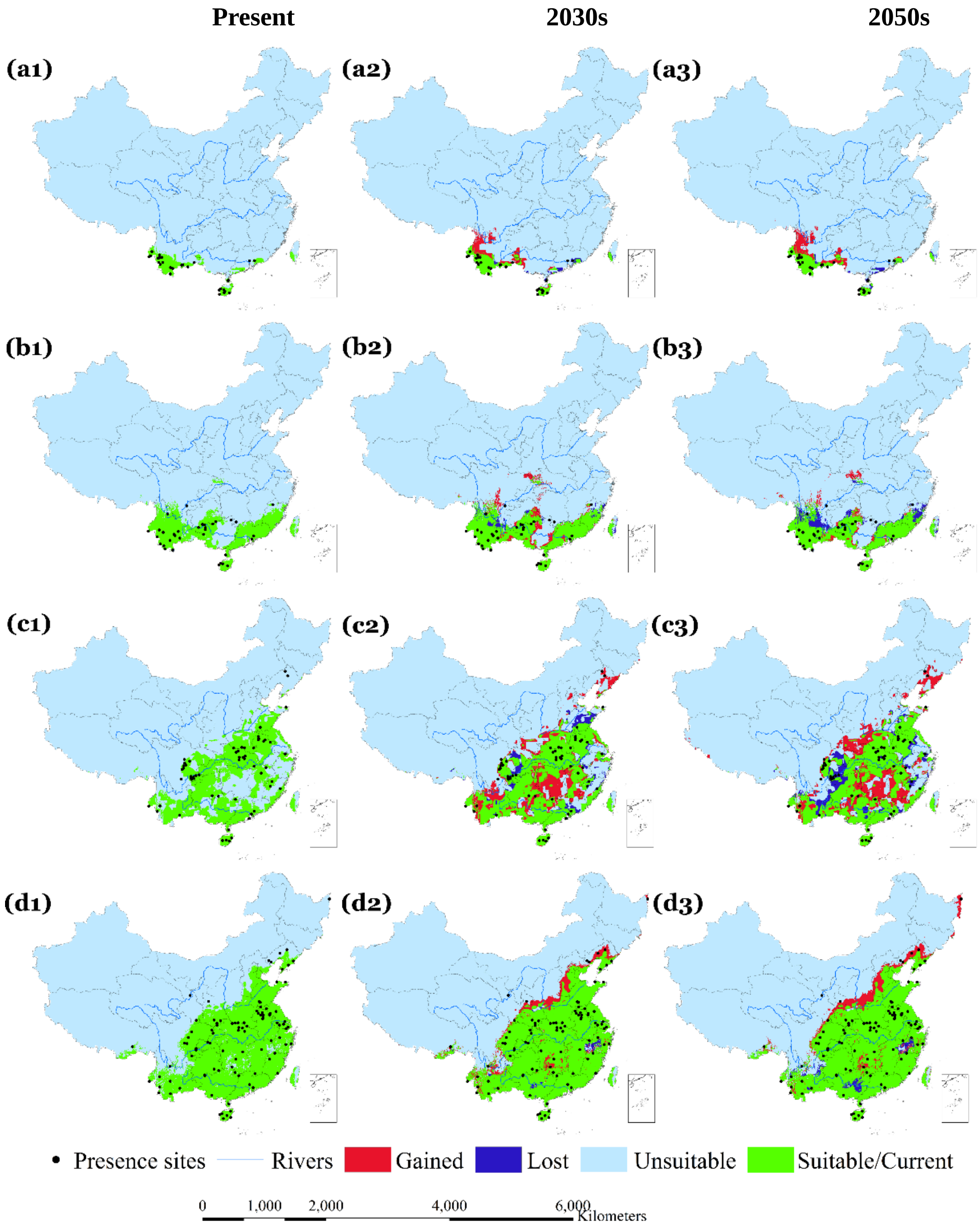

Supplementary Figure S8. Potential current (suitable and unsuitable) and future (suitable/stable, lost, gained and unsuitable) ESA for *An. dirus* (a1-a3), *An. minimus* (b1-b3), *An. lesteri* (c1-c3), *An. sinensis* (d1-d3). Future predictions are based on **CCCma_CanESM2** climate model for the 2030s and 2050s (**RCP4.5**). The second and third column indicate the 2030s and 2050s, respectively. The black dots indicate occurrence localities of four malaria vectors currently. The green color shows stable suitable areas, blue shows lost ESA and red shows gained ESA. All the lost and gained areas were calculated based on current distribution as reference. Maps created in ArcGIS 10.2 (Environmental Systems Resource Institute, ArcMap Release 10.2, ESRI, Redlands, California).

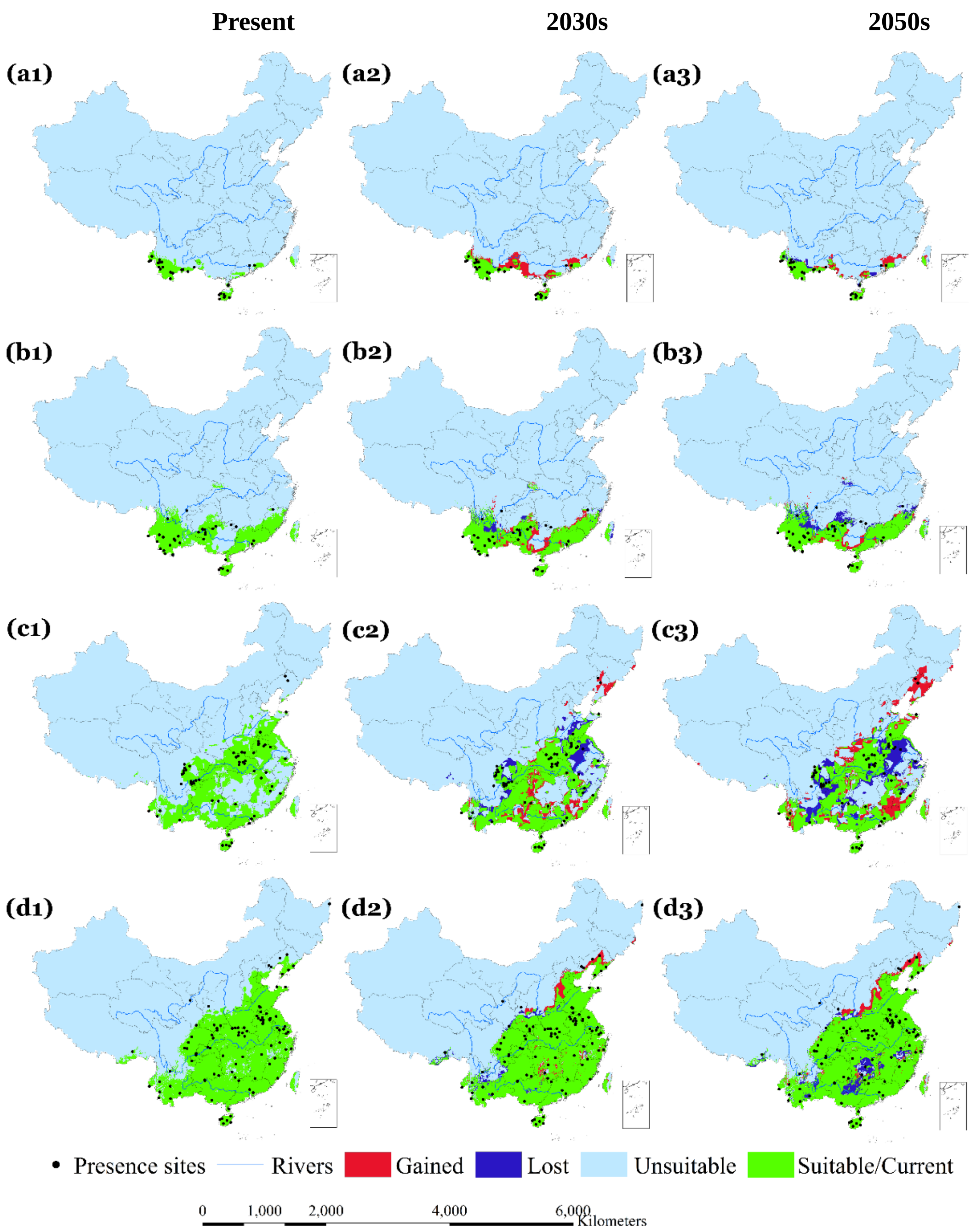

Supplementary Figure S9. **Potential current (suitable and unsuitable) and future (suitable/stable, lost, gained and unsuitable) ESA for *An. dirus* (a1-a3), *An. minimus* (b1-b3), *An. lesteri* (c1-c3), *An. sinensis* (d1-d3).** Future predictions are based on **CSIRO-Mk3.6.0** climate model for the 2030s and 2050s (**RCP4.5**). The second and third column indicate the 2030s and 2050s, respectively. The black dots indicate occurrence localities of four malaria vectors currently. The green color shows stable suitable areas, blue shows lost ESA and red shows gained ESA. All the lost and gained areas were calculated based on current distribution as reference. Maps created in ArcGIS 10.2 (Environmental Systems Resource Institute, ArcMap Release 10.2, ESRI, Redlands, California).

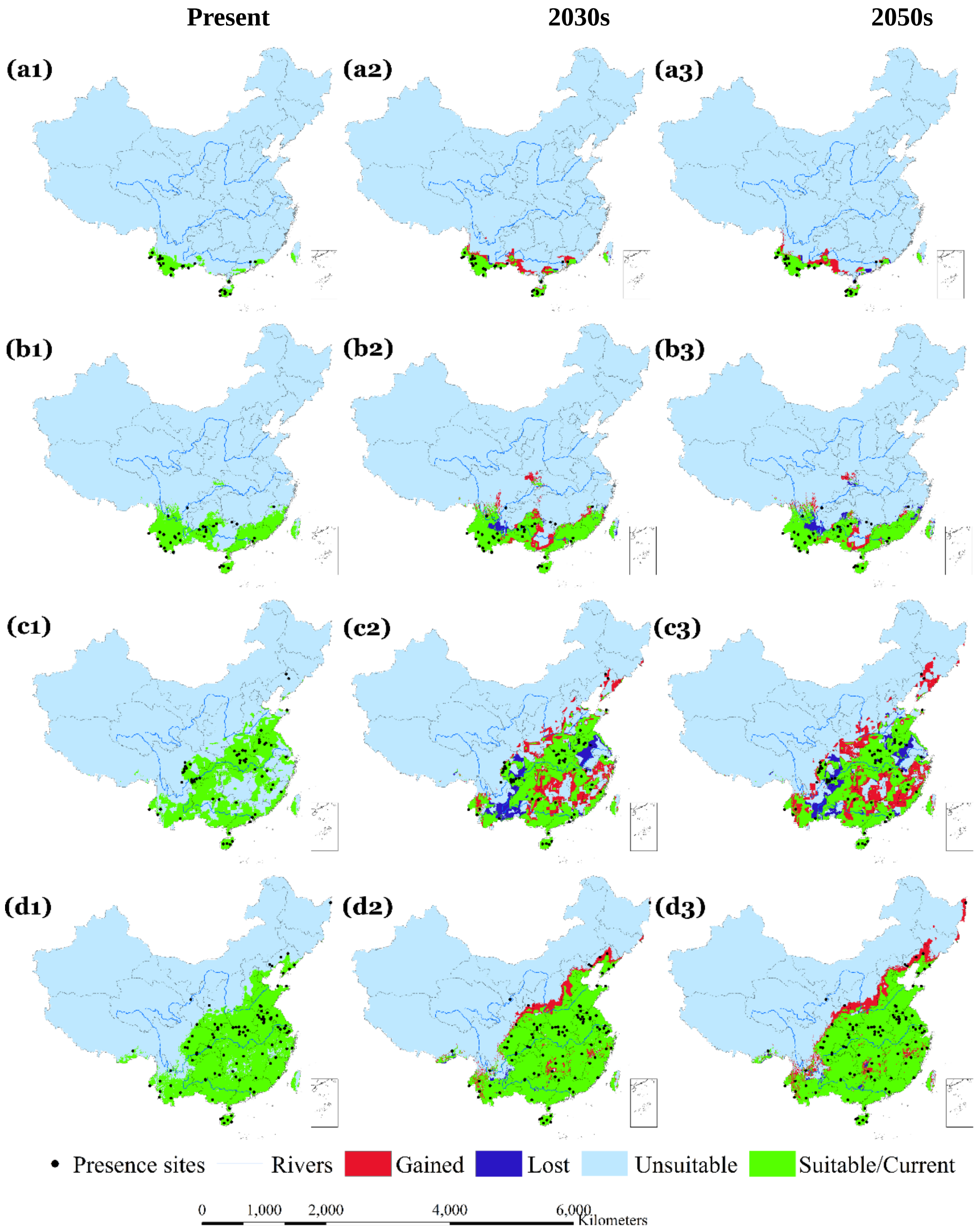

Supplementary Figure S10. Potential current (suitable and unsuitable) and future (suitable/stable, lost, gained and unsuitable) ESA for *An. dirus* (a1-a3), *An. minimus* (b1-b3), *An. lesteri* (c1-c3), *An. sinensis* (d1-d3). Future predictions are based on **BCC-CSM1-1** climate model for the 2030s and 2050s (**RCP8.5**). The second and third column indicate the 2030s and 2050s, respectively. The black dots indicate occurrence localities of four malaria vectors currently. The green color shows stable suitable areas, blue shows lost ESA and red shows gained ESA. All the lost and gained areas were calculated based on current distribution as reference. Maps created in ArcGIS 10.2 (Environmental Systems Resource Institute, ArcMap Release 10.2, ESRI, Redlands, California).

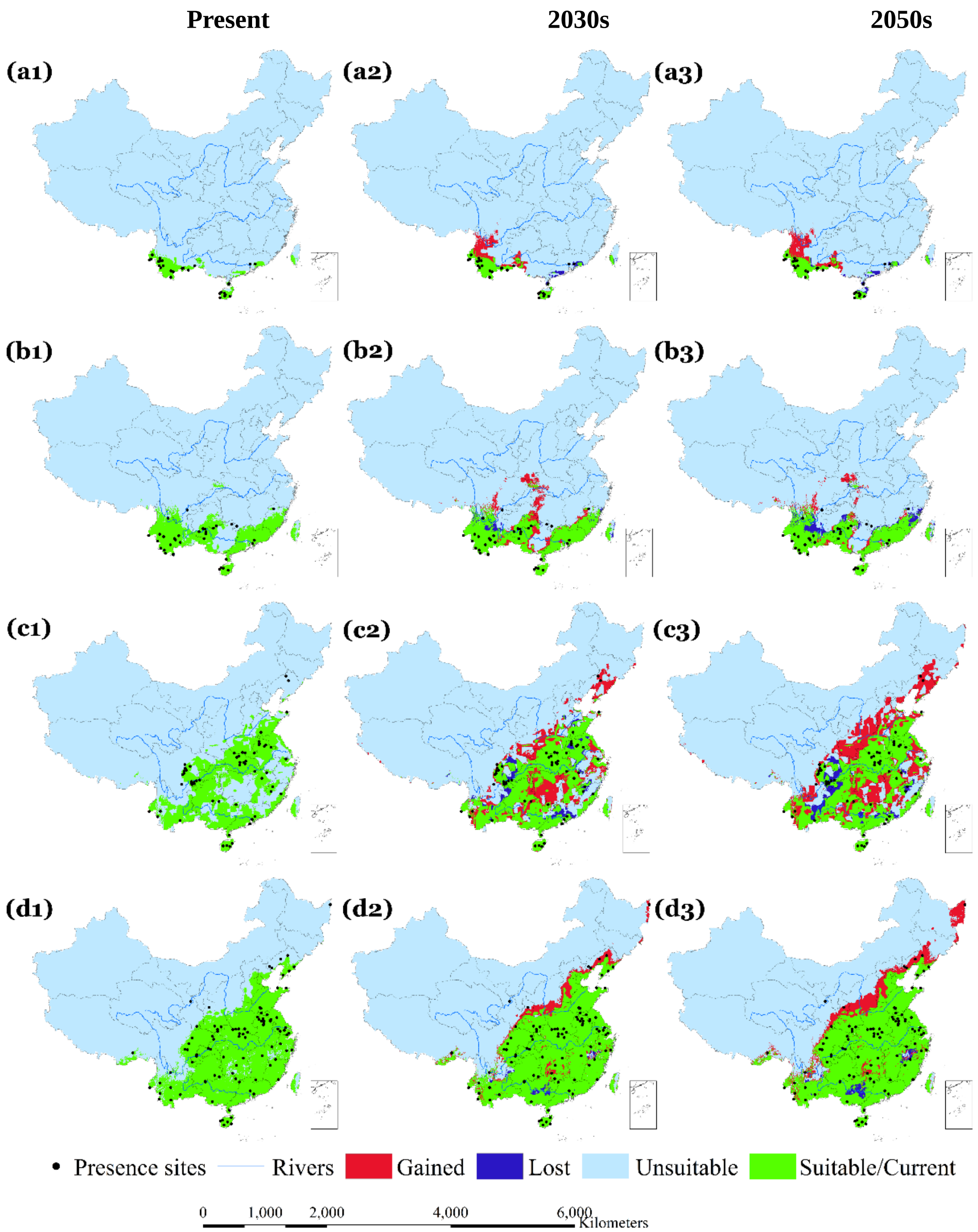

Supplementary Figure S11. Potential current (suitable and unsuitable) and future (suitable/stable, lost, gained and unsuitable) ESA for *An. dirus* (a1-a3), *An. minimus* (b1-b3), *An. lesteri* (c1-c3), *An. sinensis* (d1-d3). Future predictions are based on **CCCma_CanESM2** climate model for the 2030s and 2050s (**RCP8.5**). The second and third column indicate the 2030s and 2050s, respectively. The black dots indicate occurrence localities of four malaria vectors currently. The green color shows stable suitable areas, blue shows lost ESA and red shows gained ESA. All the lost and gained areas were calculated based on current distribution as reference. Maps created in ArcGIS 10.2 (Environmental Systems Resource Institute, ArcMap Release 10.2, ESRI, Redlands, California).

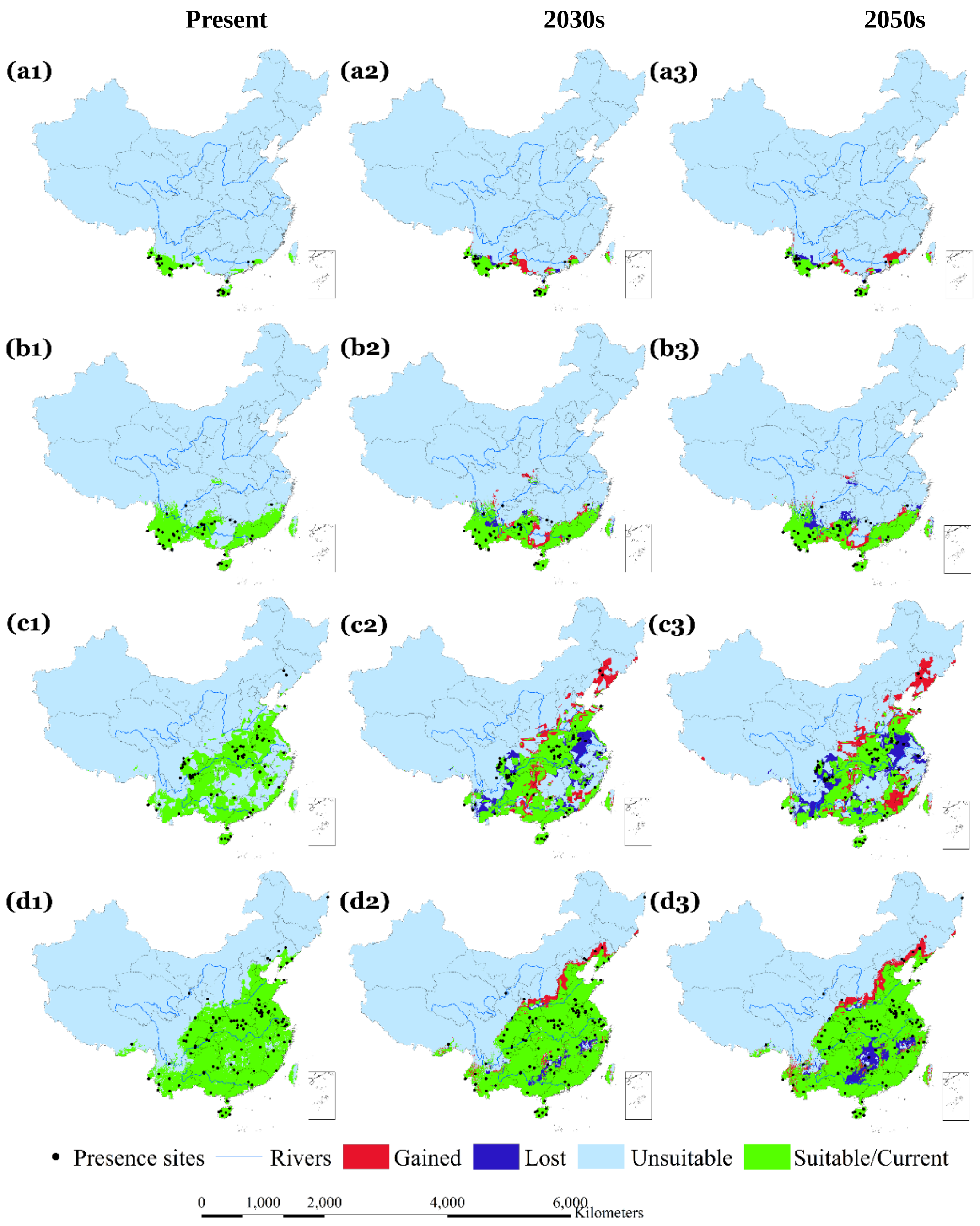

Supplementary Figure S12. Potential current (suitable and unsuitable) and future (suitable/stable, lost, gained and unsuitable) ESA for *An. dirus* (a1-a3), *An. minimus* (b1-b3), *An. sinensis* (d1-d3), *An. lesteri* (c1-c3). Future predictions are based on **CSIRO-Mk3.6.0** climate model for the 2030s and 2050s (**RCP8.5**). The second and third column indicate the 2030s and 2050s, respectively. The black dots indicate occurrence localities of four malaria vectors currently. The green color shows stable suitable areas, blue shows lost ESA and red shows gained ESA. All the lost and gained areas were calculated based on current distribution as reference. Maps created in ArcGIS 10.2 (Environmental Systems Resource Institute, ArcMap Release 10.2, ESRI, Redlands, California).

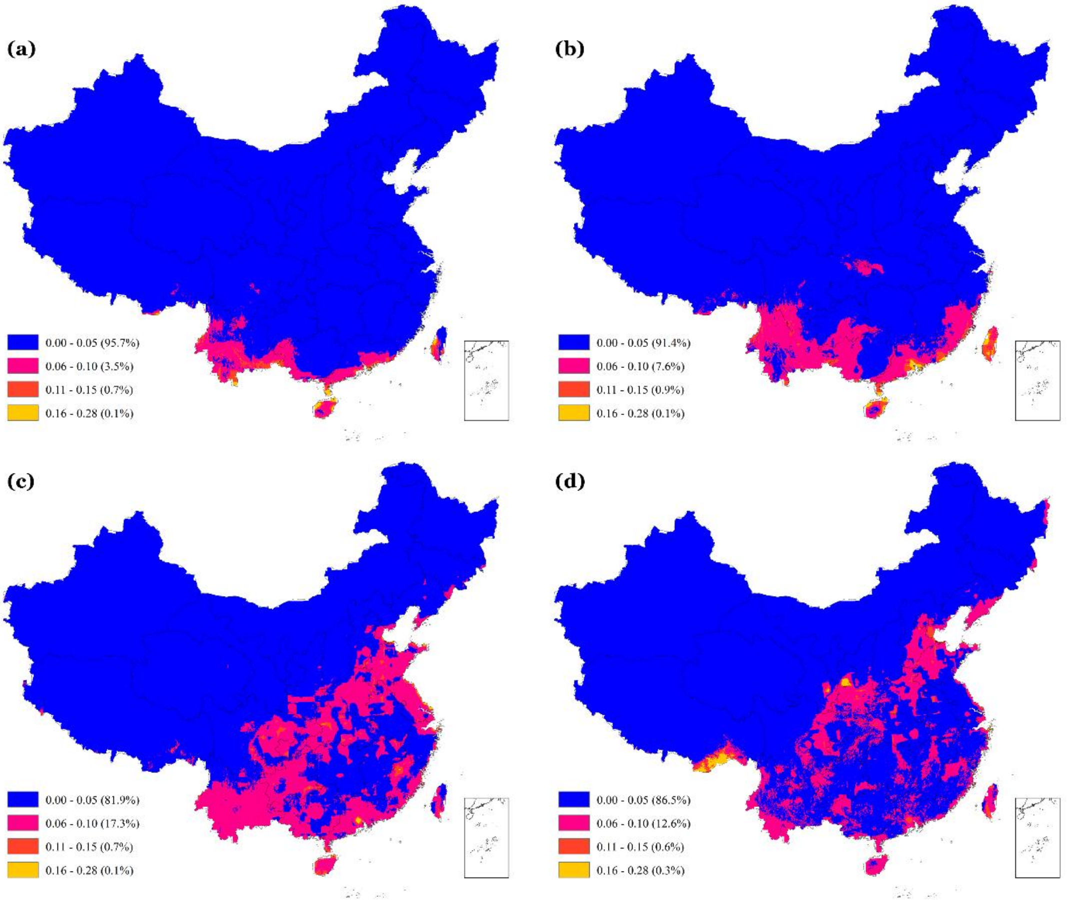

Supplementary Figure S13. Map of standard deviation of probability of presence derived from 100 replicates. (a) *An. dirus*; (b) *An. minimus*; (c) *An. lesteri*; (d) *An. sinensis*. Maps created in ArcGIS 10.2 (Environmental Systems Resource Institute, ArcMap Release 10.2, ESRI, Redlands, California).
